# Systems analysis of iron metabolism: the network of iron pools and fluxes

**DOI:** 10.1186/1752-0509-4-112

**Published:** 2010-08-13

**Authors:** Tiago JS Lopes, Tatyana Luganskaja, Maja Vujić Spasić, Matthias W Hentze, Martina U Muckenthaler, Klaus Schümann, Jens G Reich

**Affiliations:** 1 Max-Delbrueck-Centrum of Molecular Medicine, D-13092 Berlin-Buch, Germany; 2 European Molecular Biology Laboratory (EMBL), Meyerhofstr. 1, D-69117 Heidelberg, Germany; 3 Molecular Medicine, University of Heidelberg, Im Neuenheimer Feld 153, D-69120 Heidelberg, Germany; 4 Research Center for Nutrition and Food Sciences, Am Forum 5, D-85350 Freising-Weihenstephan, Germany

## Abstract

**Background:**

Every cell of the mammalian organism needs iron as trace element in numerous oxido-reductive processes as well as for transport and storage of oxygen. The very versatility of ionic iron makes it a toxic entity which can catalyze the production of radicals that damage vital membranous and macromolecular assemblies in the cell. The mammalian organism maintains therefore a complex regulatory network of iron uptake, excretion and intra-body distribution. Intracellular regulation in different cell types is intertwined with a global hormonal signalling structure. Iron deficiency as well as excess of iron are frequent and serious human disorders. They can affect every cell, but also the organism as a whole.

**Results:**

Here, we present a kinematic model of the dynamic system of iron pools and fluxes. It is based on ferrokinetic data and chemical measurements in C57BL6 wild-type mice maintained on iron-deficient, iron-adequate, or iron-loaded diet. The tracer iron levels in major tissues and organs (16 compartment) were followed for 28 days. The evaluation resulted in a whole-body model of fractional clearance rates. The analysis permits calculation of absolute flux rates in the steady-state, of iron distribution into different organs, of tracer-accessible pool sizes and of residence times of iron in the different compartments in response to three states of iron-repletion induced by the dietary regime.

**Conclusions:**

This mathematical model presents a comprehensive physiological picture of mice under three different diets with varying iron contents. The quantitative results reflect systemic properties of iron metabolism: dynamic closedness, hierarchy of time scales, switch-over response and dynamics of iron storage in parenchymal organs.

Therefore, we could assess which parameters will change under dietary perturbations and study in quantitative terms when those changes take place.

## Background

Iron as a trace metal is essential for almost all forms of life. Its biological role is attributable to its properties as a transition metal. It readily switches between its ferric (3+) and ferrous (2+) state and therefore serves as an essential prosthetic group in most cellular electron-transfer reactions. In addition, iron is a critical component of heme in hemoglobin and myoglobin, where it serves in oxygen binding and transport, which is essential for respiration in most animals.

The same oxido-reductive properties that make iron essential for life are also the cause of its toxicity, if the concentration of the free ions runs out of control. The ferrous ion can participate in Fenton chemistry and produce hydroxyl and lipid radicals with detrimental effects on structural constituents and metabolic functions of the cell. The eukaryotic cell is equipped with various proteins to handle iron, to secure its vital functions and to limit its toxicity. This includes proteins for iron uptake (metal transporter, transferrin receptors), its transport in the plasma (transferrin), and its non-toxic storage and sequestration (as ferritin). Iron metabolism is therefore interlaced with the metabolism of these proteins (reviewed in [1]).

The vital and destructive roles of iron are reflected in its tight regulation and the narrow leeway of fine-tuning in cellular subsystems. The molecular arsenal as well as the dynamic range of iron metabolism is remarkably well conserved in mammalian species. Quantitative data, scaled to body size, are surprisingly similar between, for instance, humans and mice, certain exceptions notwithstanding. The levels of variables extend over several decadic orders in a hierarchy of dynamic modes. Duodenal iron uptake is meticulously poised within a very narrow limit. As another step towards this end, the body recycles iron from degraded fractions such as erythrocyte hemoglobin. This establishes turnover rates as an additional multi-level hierarchy within the system (reviewed in [2]).

Mammalian iron metabolism has been intensively studied for over 70 years, with the fundamental paper by McCance and Widdowson [3] being among the earliest reports. These comprised the iron content of cells and organs and characterized biochemical fractions defined by ionic state and the nature of carrier proteins. In later years the distribution kinetics of tracer isotopes yielded insight into the dynamic turnover of iron fractions in organs and the whole organism. The molecular "machinery" of proteins involved in iron handling was stepwise revealed during the 1990 s with the identification of iron carrier proteins and the genes encoding them. Thus, a global understanding of iron biology based on cellular and molecular events is coming into perspective.

Certain features of iron metabolism make it a suitable subject for systems analysis aiming at integration of cellular and systemic dynamics and regulation. The cellular fate of iron is handled by a relatively small set of specialized receptor-, exporter-and storage proteins. Though some overlap with the handling of other trace metals was described, iron handling by corresponding proteins does not involve too many overlapping cellular processes. This makes systemic integration less difficult than in many other fields of biochemistry. Iron can be measured by specific chemical and physical methods, and the availability of isotopes has made dynamic studies possible. The regulation in iron deficiency and iron overload as well as after transgenic perturbation in animal models may be characterized.

A theoretical description of mammalian iron biology focuses on two complementary aspects of systems analysis: 1.) the phenomenology of what is going on in a living system, i.e. to deal with concentrations and fluxes in functionally different or disturbed states of the body. 2.) To deal with the regulatory structure that brings about these phenomena.

The mouse has become the model animal of choice for studying systemic and cellular iron metabolism. The method of genetic knock-out, knock-in, and forced expression of specific genes and the possibility to target such effects to certain cell types (Cre/Lox technology) made it possible to study the regulatory cross-talk within cells and between cells and organs in greater detail. It allows mimicking physiological differences and pathological states in the intact animal rather than in isolated cells.

A phenomenological description of internal iron kinetics has been previously published for the mouse strain C57BL/10ScSnPh [4]. The authors administered radioactive ferrous citrate into the tail vain of healthy adult animals on an iron-adequate diet and followed the distribution of the tracer into bones, spleen and liver. Their mathematical model consists of 12 iron compartments (plasma, erythrocytes, liver, extravascular fluid, body periphery, excretion compartment, and 3 *ad-hoc *compartments each in bone marrow and in spleen), and of 32 fluxes between these compartments. The authors followed the time course up to 3 weeks and provided estimated quantitative values for the 12 pool sizes and 32 fluxes with the help of ingenious "by-hand" simulation of the observed curves. Limitations of computer capacity at that time precluded the attempt to address uncertainty and redundancy of the different parameters. Therefore, many of the numerical values remained uncertain or speculative in their quantitative importance.

Mathematical models of iron kinetics were previously published for dogs [5] and for human subjects [6-8]. The parameterization in such models was derived from a detailed analysis of the time course of radioactive tracer in plasma and in erythrocytes, in one case complemented by body surface measurements [7]. The models summarized body tissues in compact compartment form (storage compartment, erythron compartment etc.), and the results of these studies became the knowledge basis for diagnostic interpretation of human diseases as it is medical practice today.

**Figure 1:**
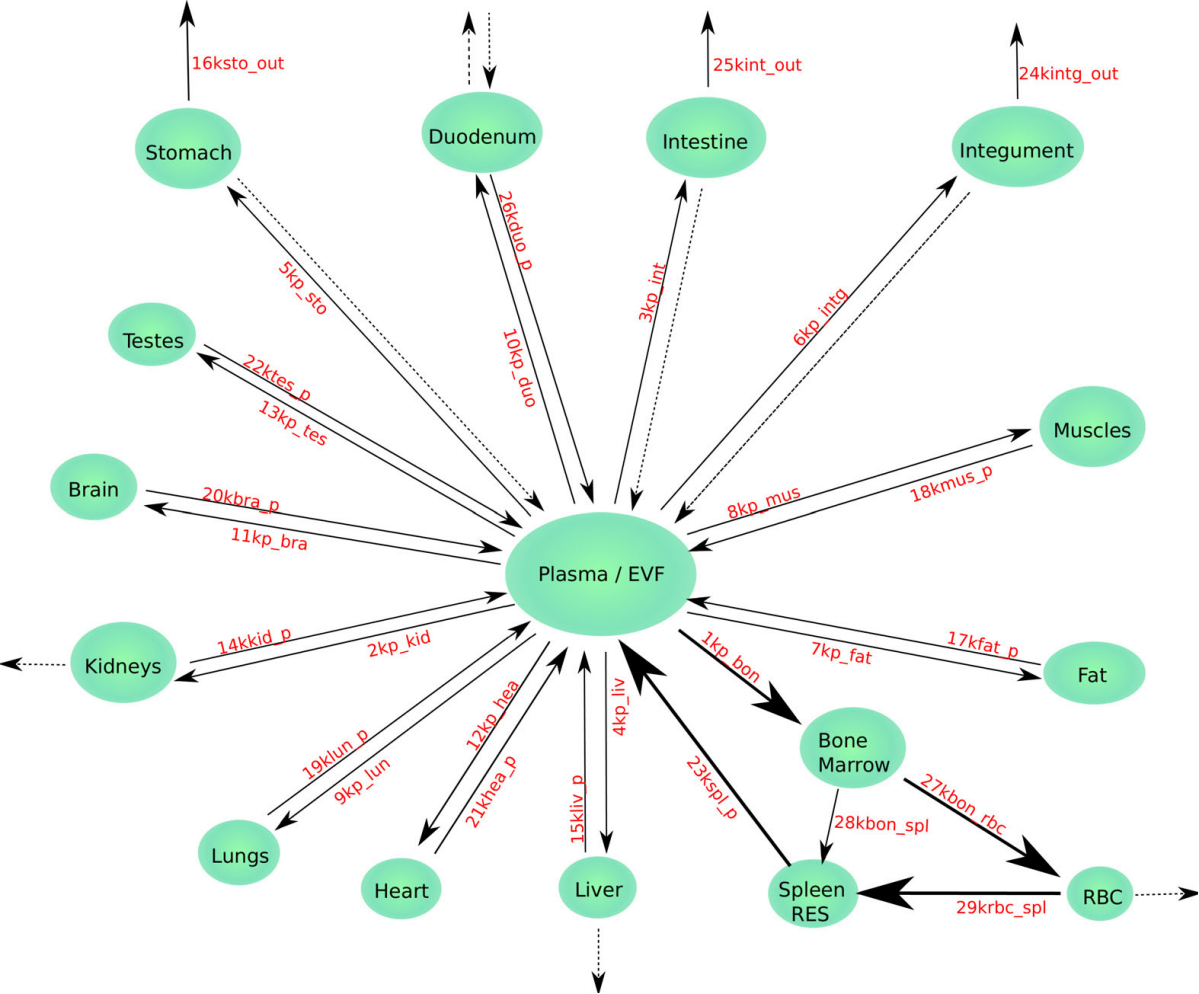
**Topological Scheme of Steady-State Iron Flux into and out of the main Body Organs of the Mouse**. All compartments receive iron from plasma/Extravascular Fluid (EVF) via the transferrin receptor endocytosis. The peripheral compartments return iron probably mainly via the ferroportin transporter. An exception is bone marrow the iron of which is rapidly channelled into haemoglobin synthesis of the maturing erythrocytes [13,59]. The depicted direct flow into the spleen represents red blood cell production (a particular feature of this organ in mice [53]) and possibly the iron uptake by spleen macrophages due to "ineffective erythropoiesis" [15]. Tracer iron bound as freshly synthesized heme may also be recycled (e.g. FLCVR-mediated) circumventing the passage through the vast red blood cell compartment. These different fluxes cannot be distinguished in a compartment clearance model as formulated here. For the red blood cell compartment we assume a component of random elimination of cells into spleen/RES, independent of cell age. The removal of senescent cells after their life span cannot be seen in the earlier stages of pulse-labelling of the iron compartments. Some of the compartments loose iron out of the body by way of cell exfoliation or desquamation (intestinal tract, skin integument), by production of bile (liver) and urine (kidney), or by bleeding. These compartments have double exits. For the purpose of parameter estimation of steady-state ^59^Fe flux the quantitatively less important elements of these double exits have been neglected (dotted arrows). Heme flow as enterohepatic absorption-secretion cycle has not been included into the figure. It would not be visible in the tracer experiment due to onset of tracer dilution over the whole body.

In this paper we present a phenomenological model of the murine iron system in the absence of pathological deviation, but under different, well controlled conditions of iron supply in the food. Based on our systematic measurements we estimate quantitative values of rate constants for the iron flux from plasma into 15 peripheral organs of the body, estimate the recycling rate of peripheral iron, and of the kinematic properties of iron-containing compartments. Figure 1 shows the model layout with its compartments and the transfer of iron between them. The dynamic and static properties of iron metabolism will be characterized in terms of systemic homeostasis. It is intended to use this detailed flux and pool model as basis for the study of the iron status of targeted genetic variants of the C57BL6 mouse strain.

## Results

### Tracer uptake into murine organs

**Figure 2:**
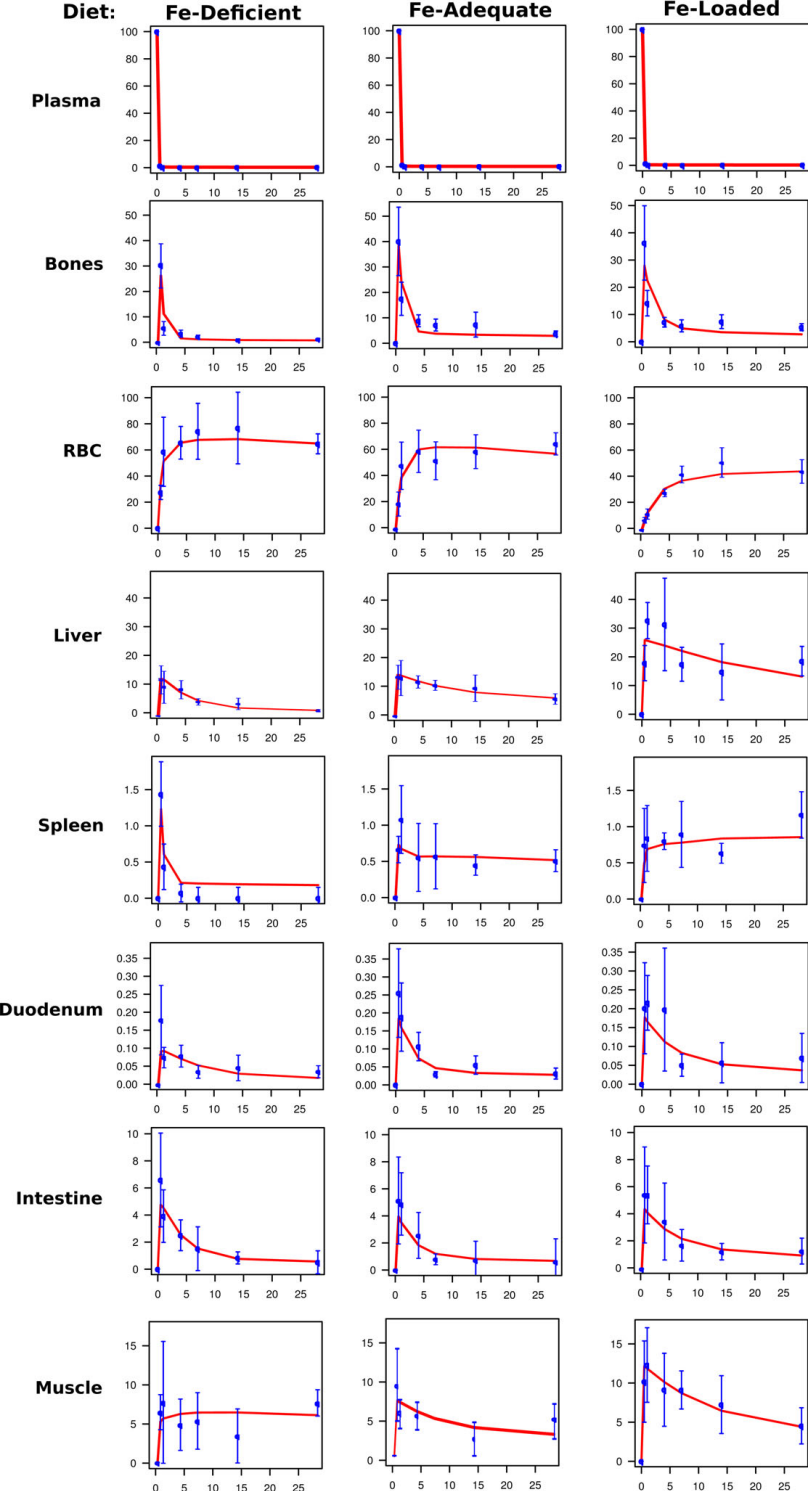
**Distribution of radioactive iron over body compartments in mice raised on diets different in iron content**. Measured values are given as mean with standard deviation. The best fit obtained by parameter estimation is drawn as continuous curve. All data were normalized to initial dose of radioactive tracer (100%), with allowance made for a slow total excretion (see methods). The kinetics of plasma clearance is very rapid (characteristic time about 60 to 100 min) and therefore not visible in detail on the scale applied here.

**Figure 3:**
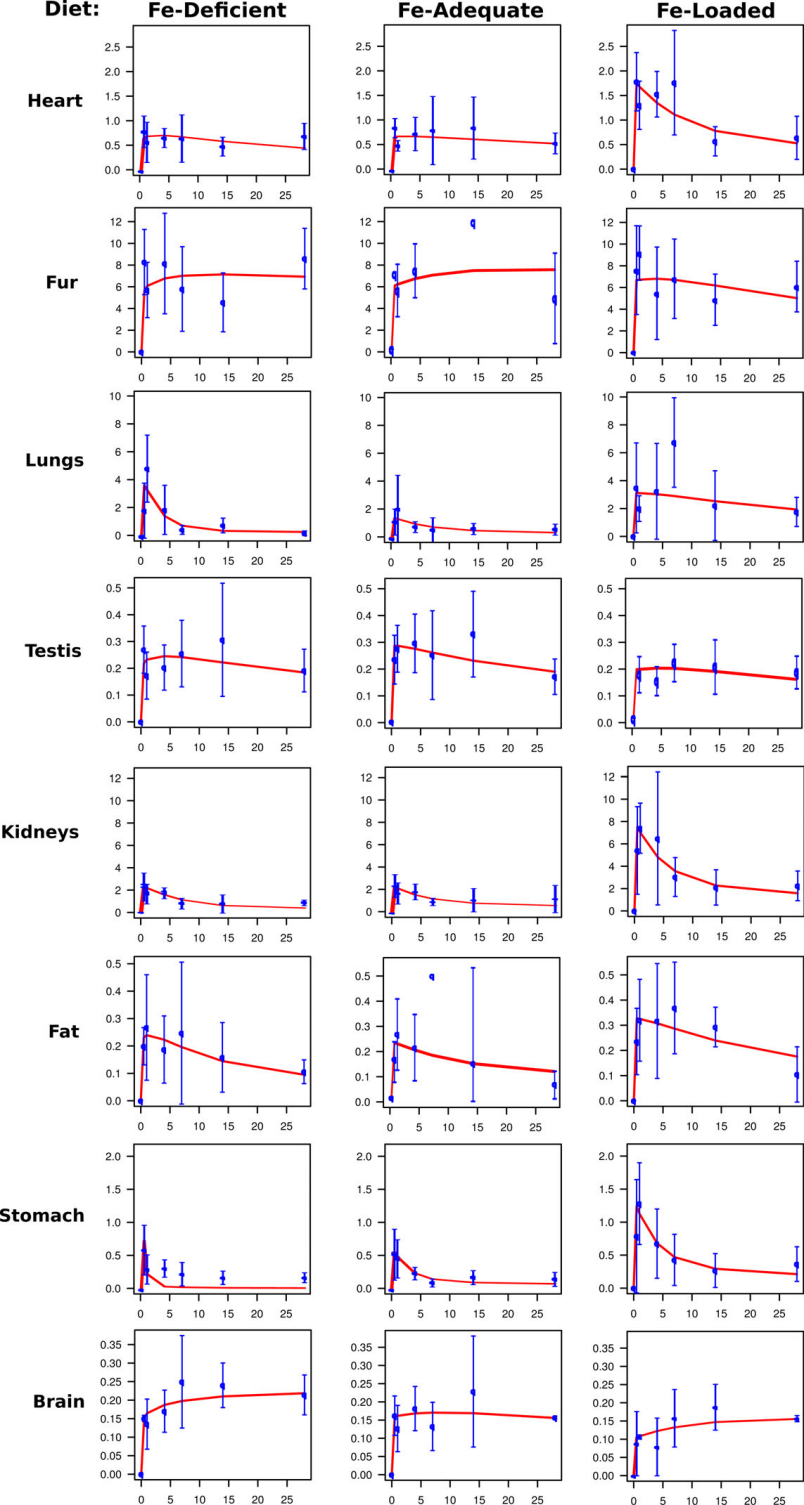
**Distribution of radioactive iron over body compartments in mice raised on diets different in iron content (continued)**. For explanation, see figure 2.

We use protocols of experiments on mice of the C57B6 strain [9]. The main tissue-related data are summarized in the Additional file 1: Table S1 to S3. They may be scaled and recalculated to represent the state of the whole organism, using the organ weights (Additional file 1: Table S4). The experimental data were obtained from iron-deficient, iron-adequate, and iron-loaded mice [9]. Additional file 1: Table S5 to S7 give the calculated organ tracer contents at different time-points after administration, scaled to the whole body. Figures 2 and 3 show a synopsis of the system's behaviour under these conditions. The measured radioactivity in the organs is given as mean plus-minus standard deviation along with a simulated time course of the "best fit" (table 1). The coefficient of variation in these data was in the range of 30%. This is common in iron studies.

**Table 1:** Parameters values, scatter intervals and best fits.

		Fe Deficient			Fe Adequate			Fe Loaded	
Parameter	Best Fit	Lower Limit	Upper Limit	Best Fit	Lower Limit	Upper Limit	Best Fit	Lower Limit	Upper Limit
1kp_bon	13.22	12.47	13.63	12.67	12.01	13.26	6.92	6.01	7.09
2kp_kid	0.42	0.28	0.54	0.45	0.36	0.51	1.62	1.18	1.82
3kp_int	0.98	0.77	1.06	0.9	0.63	1.1	0.93	0.66	1.01
4kp_liv	2.27	1.83	2.54	2.61	2.28	2.9	5.25	4.25	5.73
5kp_sto	0.09	0.06	0.18	0.12	0.08	0.17	0.27	0.21	0.37
6kp_intg	1.04	0.89	1.32	1.14	0.96	1.35	1.33	1.05	1.5
7kp_fat	0.04	0.03	0.05	0.05	0.04	0.05	0.066	0.051	0.075
8kp_mus	0.96	0.8	1.23	1.49	1.31	1.8	2.52	2.06	2.75
9kp_lun	0.79	0.64	1.06	0.31	0.22	0.44	0.63	0.56	0.75
10kp_duo	0.02	0.01	0.15	0.04	0.03	0.06	0.038	0.027	0.05
11kp_bra	0.03	0.03	0.03	0.03	0.03	0.04	0.021	0.019	0.022
12kp_hea	0.11	0.1	0.14	0.13	0.12	0.15	0.36	0.31	0.38
13kp_tes	0.04	0.04	0.05	0.06	0.05	0.06	0.043	0.037	2.68
14kkid_p	0.2	0.11	0.32	0.2	0.14	0.25	0.23	0.16	0.32
15kliv_p	0.25	0.21	0.32	0.14	0.11	0.16	0.1	0.073	0.12
16ksto_out	0.18	0.09	1.72	0.37	0.27	0.49	0.29	0.2	0.4
17kfat_p	0.1	0.06	0.19	0.13	0.1	0.15	0.099	0.079	0.12
18kmus_p	0.03	0.02	0.05	0.15	0.12	0.21	0.14	0.11	0.17
19klun_p	0.41	0.37	0.52	0.19	0.11	0.3	0.086	0.065	0.12
20kbra_p	0.02	0.02	0.03	0.06	0.05	0.07	0.028	0.022	0.034
21khea_p	0.06	0.03	0.08	0.08	0.06	0.09	0.17	0.14	0.19
22ktes_p	0.05	0.03	0.07	0.09	0.07	0.12	0.067	0.044	7.16
23kspl_p	14.61	13.86	15	7.29	5.53	9.15	1.91	1.52	2.33
24kintg_out	0.03	0.02	0.05	0.04	0.03	0.06	0.072	0.057	0.102
25kint_out	0.3	0.22	0.4	0.36	0.26	0.42	0.22	0.16	0.26
26kduo_p	0.17	0.12	2.55	0.42	0.32	0.55	0.24	0.18	0.34
27kbon_rbc	1.85	1.74	1.92	1.07	0.93	1.26	0.5	0.48	0.57
28kbon_spl	0.56	0.4	0.83	0.1	0.08	0.13	0.046	0.033	0.058
29krbc_spl	0.03	0.02	0.04	0.06	0.05	0.07	0.032	0.027	0.047
fval chi_sqr (fit criterion)	0.58			0.72			1.05		
sq root of mean weighted squared dev (fit quality)	0.07			0.08			0.07		

The parameter estimates (expressed per day) were obtained by an optimization algorithm that minimized the fit criterion as defined in the paragraph "parameter estimation strategy" of methods section. Rate parameters were estimated under the assumption of approximate steady-state fluxes. Displayed are the best-fit values. The statistical limit of acceptable parameter value was obtained by perturbing the tracer measurements at random (equidistributed, "Monte-Carlo resampling") around the mean within the standard deviation, both displayed in Additional file 1: Table S1 to S3.

The value of the total plasma iron clearance rate had to be prescribed as fixed value in order to avoid excessive parameter correlation (see paragraph "parameter identifiability"). Their value was chosen to the convenient value of 20 per day, approximately in keeping with the data of Trinder et al. [10] and Brodsky et al.[53]. Any other choice of the total clearance rate will affect all out-of-plasma estimates in the table proportionally.

The criterion of best fit "fval chi_sqr" was selected from an analysis of the data structure (see "fit criterion" in section methods).

"sq root of mean weighted squared dev" (see section "quality of final fit" in methods) is a measure of a typical deviation between measurement and optimal fit.

### Estimation of clearance rate parameters

The estimation of the rate parameters requires tackling with the mathematical difficulty of data fluctuation and parameter redundancy in this multi-level time hierarchy (see methods). The resulting uncertainty of parameter estimates, after elimination of parameter interdependence, was in the range of the data scatter. Table 1 contains the estimated fractional turnover rates for all fluxes shown in figure 1, with estimates of their statistical fluctuation obtained by Monte-Carlo resampling of the data protocol (see methods). Additional file 2: Figure S1 contains the "best" estimates of the fractional turnover rate constants, and Additional file 2: Figure S2 displays the relative distribution of plasma iron turnover fluxes into periphery. The "fit quality" addressed in table 1 is a global measure of the deviation between model and data. A value of 0.07-0.08 is satisfactory.

### Calculation of flux rates of iron exchange between the central and peripheral compartments

The system of equations to predict the "best fit" is an idealized summary of tracer dynamics. It can be used to calculate the distribution dynamics of steady-state flux rates of transferrin-bound iron into and out of the various organs (Additional file 2: Figure S3, Additional file 1: Table S8).

### Plasma iron pool

The transferrin-bound iron pool in plasma is in the range of 1-2 μg, see Additional file 1: Table S9). This is small compared to all other pools. It equilibrates tracer iron with the internal iron within minutes. There is another pool of similar size in the transcapillary vascular fluid [12] which equilibrates with a rate constant of about 1d^-1^. Since our data do not contain ferrokinetic measurements of the extravascular pool, we decided not to introduce it as a separate compartment of the model.

### Total plasma clearance of iron

The total clearance rate of transferrin-bound plasma iron is given by the sum of fractional clearance rates into the periphery (table 1). Its absolute value is not accessible from our data set, because the first time point was 12 h, i.e. when the clearance was nearly complete. Precise determination of plasma clearance would require repeated blood sampling and measurement of plasma radioactivity in each animal at very short intervals after injection (first hour). Cavill et al. [29,30] have summarized the corresponding diagnostic problems in humans. Such procedures are not feasible in mice. Therefore, we used a rough estimate of plasma iron clearance taken from 2-h points in the murine experiments with a diet design similar to ours [10]. They permit the calculation (Additional file 1: Table S9) of the approximate total clearance rate of the plasma iron pool. The estimate from 2-h values may be somewhat too low due to early reflux of label iron via bone marrow and spleen into the plasma. Based on other literature data we introduced a rounded total fractional clearance of 20 daily turnovers of the plasma pool. This was a set value in the parameter estimation and defines the time scale for all other iron data. Different values, higher or lower, would affect the values in Additional file 2: Figure S1 and Additional file 1: Table S8 proportionally, but not the relative values of iron distribution into tissues and organs as shown in Additional file 2: Figure S2.

### Fractional iron clearance into the peripheral compartments

Table 1 lists and Additional file 2: Figure S2 depicts the fractional clearance parameters of iron into the various organs. The iron content of the transferrin pool increases with the iron content of the diet (Additional file 1: Table S9). The total clearance constant of plasma iron, however, remains nearly unchanged (see Additional file 1: Table S9).

### Iron uptake into the erythropoietic system

The bulk of tracer outflow from plasma goes into the erythropoietic bone marrow and is rapidly incorporated into hemoglobin [13,14]. About 63% of plasma iron is cleared into bone marrow in iron-adequate mice and practically the same fraction (66%) in iron deficiency. This fraction is somewhat lower than in other species (e.g. [2,14,31]). In iron-rich mice it decreased to about half of that (35%) under steady-state conditions. However, corresponding plasma iron turnover rates increase only by 22% (figures 2 and 5 of [10]). Therefore, the absolute rate of iron flow to the erythron is reduced in iron-loaded condition (from 19 to 14 μg/body/day, see Additional file 1: Table S8). Switching the system into the storage mode [32] appears to overshoot and reduce the influx into the erythrocyte-forming pathway. Slightly reduced hemoglobin contents were observed accordingly [9].

### Flux rates through compartments and size of tracer-accessible peripheral pools

Based on the iron concentration in plasma (Additional file 1: Table S9) and fractional clearance parameters (Additional file 2: Figure S2 and table 1) it is possible to calculate the absolute flux rates for iron from the plasma into the connected compartments (eq. 5; see Additional file 2: Figure S3, Additional file 1: Table S8). Dividing steady state iron flux rates into a given compartment by the fractional clearance rate of that compartment gives an estimate of the "tracer-accessible" pool size (calculation not possible for duodenum). Additional file 1: Table S10 and Additional file 2: Figure S4 show that there are 4 scales of iron pool (see discussion below).

### Hierarchy of iron residence times in different organs

A hierarchy of iron residence times is revealed based on estimated clearance rates from the compartments (eq. 4). Additional file 1: Table S11 shows the expected average residence time of an iron molecule when it reaches a given compartment. There is a rapid circulation of iron between plasma, bone marrow and spleen (residence times below 1 day), but a spectrum of longer residence times in the other organs (intestinal segments: ~ 2-3 days; inner organs: 5 to 13 days; and erythrocytes, brain and integument: 16 days and longer). In the latter 3 organs iron may be kept for a month and longer, which would point to a complex interplay of biosynthesis, storage and export that cannot be unravelled by whole-body ferrokinetic study. In the case of rapidly renewed cells (intestine, skin) this residence time is the same as that of the whole cell, whereas in stationary cells it reflects the well-known specific renewal rate of protein within such cells [33].

### Comparison of "tracer-accessible" pools with the unlabelled non-heme iron pool

The pools accessible for radioactive labels (Additional file 1: Table S10) may be compared with the non-heme iron content of the same compartment (Additional file 1: Table S12). The latter store increases considerably with higher iron loads in parenchymal organs (liver: 16-fold, spleen: 7-fold, kidneys: 1.4-fold), whereas heart muscle and presumably other organs do not pile up such stores. By contrast, the ^59^Fe-accessible "kinetic" pools increase only 1.7 to 3.4 fold on iron load (see Additional file 1: Table S13). It also turns out (Additional file 1: Table S13) that the relative size of the tracer-labelled pool in liver and spleen drops from ~ 25% to ~ 6%, while this share is increased from ~ 20% to ~ 40% in kidney and heart.

## Discussion

### The Mathematical Model

From the viewpoint of systems analysis, iron metabolism of the body is an open black-box with input, internal processing and output. Absorption fluxes and losses are relatively slow compared to internal iron circulation [34,35]. The most relevant internal dynamic events occur within the first few days after tracer injection. They involve the plasma iron turnover and the turnover of the erythron. On this time scale the system is nearly closed, with input (absorption) and output (excretion, desquamation) being slow compared to the dominant rates of inner metabolism. Iron absorption from guts is in the range of 0.5 μg per day (calculated to whole-body scale from the data of Bahram et al. [36] and Lebeau et al. [21], whereas the rate of total plasma iron exchange with the body periphery is in the range 20-30 μg per day (Additional file 1: Table S8). Such a system will approach an inner steady-state with slowly drifting concentrations and fluxes.

To study such a steady-state one can measure stationary content variables and inject a tiny dose of ^59^Fe, preferentially bound to its transferrin carrier [37], into the central compartment of radioactive iron. In the initial hours after mixing of the injected iron, when the periphery does not appreciably return tracer, the flow of blood into the organs is proportional to the flow of tracer. This initiates distribution dynamics of the tracer in conformity with the pools and stationary rates of the unlabelled bulk of iron in the steady-state, which is not disturbed by the addition of trace amounts. The time course of the ensuing run-off of tracer distribution obeys a system of ordinary differential equations (methods and Additional file 3).

### Iron status of the adult mice on different dietary regimes

The experimental data which form the basis for the model calculations presented here are derived in adult mice that were on different dietary regimes during growth. The adult animal develops a steady-state which is maintained during its further adult life, i.e. for approximately 1-2 years. This assumption is prerequisite for the ferrokinetic model. The empirical background for the model consists of the static iron status and of tracer dynamic data.

### Modelling iron metabolism by the ^59^Fe tracer method

The literature contains numerous papers [2,5,6,8,38] which measured the dynamics of iron metabolism on the intact organism with the help of the tracer ^59^Fe. The basic rationale is that the tracer, while being always measurable as radioactivity, due to its tiny relative amount does not perturb appreciably the iron status of the body. Most of the mathematical models derived from such measurements were obtained for humans, dogs and rats. Vácha et al.[4] derived a whole-body model for the normal mouse (related to our strain), collected from measurements in blood, liver and spleen, without systematic control of dietary regime. The model was based on a number of ad-hoc assumptions of fluxes, which partly could not be substantiated by cellular mechanisms, and the parameters were in part estimated without a whole-model statistical fitting procedure (computer capacity-limited). We can confirm, with the experimental evidence now available, that their ingenious model, in spite of some speculative elements due to limited molecular knowledge, gives a remarkably adequate description of the global dynamic structure of murine iron metabolism.

### Static iron status

This is defined as the iron content of the iron fractions in the different organs and tissues. It reflects the expression of protein carriers to which iron is bound (transferrin, ferritin, heme proteins etc.). We can assume that this status is stable during the observation period.

### Dynamic fluxes

The second data type is the time course of iron flow through body organs after administration of a radioactive tracer. The data base stems from partly published measurements of our own labs [9] and from studies done elsewhere under a comparable experimental design. The aim is to integrate the entire data set into an integrative model, thereby displaying the systemic structure which is not obvious from inspection of raw data.

### Kinematic model of iron steady-state

The crucial supposition for a representative model is that iron fluxes in the body are (approximately) balanced and cellular pools do not fluctuate violently during the experimental period. Tracer injection permits to collect data on internal fluxes without upsetting the steady-state. Analysis of the fate of tracer-^59^Fe in the tissues in terms of a linear system of differential equations describing influx, outflux and internal metabolism of the system gives a picture of the prevailing "kinematics" of the system, i.e. it describes what happens, not the causes and controlling mechanisms. Such a phenomenology is the prerequisite for any in-depth systemic description.

### Inhomogeneity of compartments

For some important tissues under consideration the assumption of homogeneity is not valid. This applies to liver, which consists of parenchymal cells (hepatocytes) and cells of the RES (Kupffer-cells). Both types have different iron regulation. Similarly, the murine spleen has subcompartments, of erythropoietic and of macrophage cells. Muscle tissue contains a large fraction of iron in myoglobin, the turnover of which is different from that of the macrophages in muscle. On the whole, the macrophages of RES are spread over a multiplicity of organs and change their distribution in inflammation. For the sake of model calculation, nevertheless, we treated these organs as compartments.

### Numerical parameter estimation

Tracer motion in a steady-state system of homogenous pools (like in figure 1) can be modelled by ordinary linear differential equations. In theory, the concentration of tracer in these pools follows a time course described by superimposed exponentials. In the initially labelled central compartment (plasma) the tracer content falls monotonously. In all the other compartments, initially void of tracer, the concentration rises to a maximum and then turns into a monotonously decreasing phase of recycling into plasma together with outflux out of the body. The parameter values of the interconnected system can be obtained as "best fit" according to a suitable distance criterion. In practice this estimation process may run into two types of difficulty: statistical scatter and redundancy of the parameter space.

The scatter of measured data in most biological systems is considerable and cannot be avoided. The reason is partly individual variation between subjects and partly the technical difficulty to exactly repeat the same experiment. A statistical model of this situation can, at best, be a close approximation to the measured data in the form of an idealized curve. We repeated the estimation procedure on sets of artificially generated data which keep the error structure of the observed data. The range of parameter fluctuation was revealed in this way.

### Interdependence (correlation) of parameter estimates

This is an unavoidable problem of complex biological models. It became clear from the ACE analysis (see methods) that our data contained two causes of parameter interdependence: insufficient resolution at very early time after tracer injection, and cases of double output of tracer back into plasma and out of the body. We overcame these problems by prescribing an approximate value of the total plasma clearance calculated from the data of Trinder et al. [10]. Furthermore, we replaced double outfluxes by a single lumped one, thus not specifying the precise fractional contribution of each pathway (see dotted outflux arrows in figure 1). In this way we obtained parameter estimates with a reasonable range of scatter avoiding strong intercorrelation.

### Further parameters of the model

The set of clearance-and rate-parameters resulting from the parameterization is given in table 1, and visualized in Additional file 2: Figure S1. The quality of the fit is satisfactory, as demonstrated in figures 2, 3, 4. Table 1 contains the most compact representation of the information content of the empirical data. It can be used to calculate flux rates (Additional file 1: Table S8), pool sizes (Additional file 1: Table S10) and as well as a characteristic temporal structure of the system (Additional file 1: Table S11). Additional file 2: Figure S2 through S4 visualize these quantitative estimates. These indirectly derived data indicate scatter intervals of system-relevant parameters. Their totality is amenable to physiological interpretation of the static and dynamic state of the iron system in the mouse in the different "lifestyle" regimes studied.

### Physiological interpretation of the global system

#### Iron metabolism can be described as a closed compartment system

The quasi-closed state of the iron system together with the ensuing internal steady-state makes into possible to simplify the non-linear structure to a system of ordinary linear differential equations. The dynamics of tracer motion depicts the statics of the underlying stationary flux-and-pool network. We could build on a number of previous attempts to model iron metabolism in this way [5-8,38], reviews in [2,38]. The novel aspect here is the detailed reversible balance in a network of peripheral tissues that were previously combined *ad hoc *to black boxes.

#### Iron metabolism is organized as temporal hierarchy on five time scales

Analysis of the clearance parameters of our experiments (table 1, transformed into residence times-Additional file 1: Table S11) and of literature data [10,18,19] lead to the following grouping of characteristic time periods:

-Rapid mixing time of the transferrin-bound plasma/ECF pool: below 1 h

-Total clearance time of plasma iron due mainly to flux into bone marrow, liver and muscle: ~ 1 hour.

-Clearance time of compartments that return tracer into plasma (descending branches in figure 3, and Additional file 2: Figure S1): between ~2 days in the intestinal tract and ~8 days in parenchymal organs.

-Incorporation time into iron-carrying proteins in red blood cells, muscles, integument or fat: ~ 1 month.

-Characteristic life-time of iron molecules in the whole body: approaches 200 days in the adult mouse. If the mouse has acquired a store in the adolescent stage (adequate diet), this would be sufficient for its whole life. It is therefore difficult to induce iron-depletion anemia in the mouse (contrast to humans).

The time hierarchy does not change appreciably between different iron statuses in normal mice (confirming the conclusion in [10]).

#### Iron turnover in the plasma compartment depends on the iron status

The concentration of transferrin-bound plasma iron in plasma is in the range of 100-200 μg/dL in the mouse (Additional file 1: Table S9). This is similar to other mammalian species (e.g. [12,39-42]). The iron concentration tends to lower values in iron-deficient and to higher values in iron-loaded mice. The iron clearance from plasma defines a half-time of renewal in the range of 1-2 hours, again similar for species otherwise as different as *Mus musculus *and *Homo sapiens*. Rats [41] and dogs [5,39] are also in the same range. In rats, however, iron deficiency does not lower the plasma concentration [41].

#### Iron distribution into body periphery is a three-level hierarchy of flux rates

The initial tracer concentration in plasma becomes rapidly cleared within a few hours after administration and stays at a low, but steady value afterwards. This coincides with the ascending tracer curves in the peripheral compartments (figures 2 and 3. The initial distribution is complete at the first time of measurement (12 h). The position of the maximum fixes the time point when plasma tracer is nearly washed out and the periphery begins to return some of the previously accumulated tracer iron into the plasma (figures 2 and 3. The continuous decrease of organ tracer content begins after 12-24 hours. It is an expression of the fact that "fresh" cellular iron is not only stored or channelled into biosynthesis, but also shows an appreciable back-flow into the plasma.

The descending branch of the peripheral tracer curves show that all tissues return the radioactivity into the plasma, unless they lose it by desquamation, which is the case for intestine and integument. This characteristic pattern proves that iron flux into the periphery and reflux into plasma take place simultaneously.

The quantitative level of all the superimposed fluxes can result only from a deeper analysis of the corresponding mathematical model. This analysis yields a set of fractional clearance parameters (table 1). From these values and by application of the steady-state assumption all iron fluxes can be estimated when the iron content of the central compartment is available. Data by Trinder et al. [10] contribute an estimate of the total plasma turnover clearance rate (Additional file 1: Table S9).

#### Three clusters of flux rates may be distinguished

-flux through the erythron (range of 10 to 20 μg/day per mouse)

-flux through peripheral compartments with storage function (liver, muscle, integument, intestinal tract, kidneys, lungs, heart: 0.5 to 4 μg/day per mouse)

-flux through organs with slow iron turn-over (testicles, fat, brain) -0.05-0.08 μg per day per mouse.

Share of flux into tissues mirrors transferrin receptor expression

The clearance time of plasma iron is in the range of 1 h, largely independent of the plasma iron content and hence the state of the animal's iron supply. This linear kinetics suggests that the total population of TFR1 receptor molecules (responsible for most of the iron uptake) works far below its maximal capacity in all cells. The share of radioiron going into the body organs reflects this tissue-specific transferrin receptor expression. In contrast to the rather stable total clearance time the share of radioiron is dependent on the physiological state (Additional file 2: Figure S2). In the states of iron depletion and of normal iron supply more than two thirds of the plasma iron turnover is directed to the erythropoietic bone marrow and is rapidly incorporated into hemoglobin. This is, again, similar to other species [2,13,14,31]. The corresponding fraction of tracer iron passes through the immature cells of the erythropoietic lineage until it reaches the erythrocyte compartment.

#### Tracer distribution iron-rich condition reflects the switch-over to the storage mode

The flux through the storage pathway into parenchymal organs increases from 25% to 49% of plasma turnover (table 1; Additional file 1: Table S8; visualized in Additional file 2: Figure S2 and S3). Stores are filled up in liver, kidney, spleen/RES (Additional file 1: Table S12), to a lesser extent also heart and skeletal muscle, but not integument and brain.

#### Tissue cells equilibrate influx and reflux of iron to maintain the iron pool

An adult mouse does not grow much during its life-time of ~2 years (if not killed before). Iron is taken up by cells with a time characteristic of a few days and must, therefore, be balanced by corresponding iron-release. Muscle, fat, heart, lungs, brain and testicles excrete iron into plasma or extravascular fluid. The influx of tracer is mediated by transferrin receptor. It is not clear from tracer data whether the export is mediated only by the ferroportin channel [11], or also via catabolism of heme-bound iron. Ferroportin is dominantly expressed in liver, duodenum, and macrophages, and to a lesser extent also in other tissues [43]. Ferroportin is not involved in the case of loss of whole cells (erythrocytes, intestine, and integument). The tracer data as used here cannot distinguish between export of iron and loss of whole cells. They yield only an estimate of the total flux out of the compartment.

#### Intracellular residence time of iron is longer than the life time of its protein "carriers"

The life-time of the iron-storage proteins (such as apoferritin/holoferritin) is in the range of one day in the liver [33]. Up to 4500 iron ions can be stored in one ferritin molecule [44], and become released on proteolytic ferritin degradation. The residence time of iron in the liver cell, however, is much longer-in the range of 1-2 weeks (Additional file 1: Table S11). This shows that iron released into the very small labile iron pool does not leave the cell, but is re-utilized. This slow export conforms to well-known data showing how slowly iron is mobilized from ferritin stores to replace iron losses, e.g. after phlebotomy (in men: [32,45]). Intracellular iron stores are no inert long-term reserves, but are continuously turned over within the cell and may therefore be directed, in accordance with changing requirements, into the three competing pathways (biosynthesis, storage, export).

#### Readily accessible tissue iron pools are a fraction of the non-heme iron

These iron pools are stored in different subcompartments, mainly in non-heme form. The iron-loaded liver stores ferritin in the hepatocytes and a less mobilizable (hemosiderin?) form in the Kupffer cell [46-48]. The labelled and unlabelled iron data from whole organs do not permit to differentiate quantitatively between parenchymal and macrophage iron in such mixed cases. Tracer dynamics identifies iron pools that become quickly labelled. Their pool sizes have been estimated from the fractional plasma iron turnover and the tissue clearance rates (Additional file 1: Table S9 and table 1). Three groups may be distinguished. Red blood cells contain as hemoglobin the largest readily labelled iron pool (~ 300 μg Fe per mouse, about 50% of total haemoglobin-iron, see calculation in Additional file 1: Table S9). There is a second cluster of pools (integument, liver, bone marrow, skeletal muscles, skin), each containing about 20-40 μg Fe. In particular the hepatic iron pool is expandable in iron overload to reach a kinetic pool level of ~ 100 μg Fe, presumably in ferritin form. A still larger store can possibly accumulate on a longer time scale, which is not covered here. There are additional pools with an iron content (lungs, kidneys, intestine, heart, and spleen) of about 3 μg Fe each, which can moderately expand up to 4-14 μg (Additional file 2: Figure S4, lower left). Other organs, such as fat, testicles or brain, are not able to store more iron in overload. Additional file 1: Table S13 shows that in some tissues the readily accessible pools are only a fraction (6 to 40%) of cellular non-heme iron.

#### There are two kinetically distinct major iron pools in the mouse body

The total tracer-accessible iron amounts to ~400 μg (Additional file 1: Table S10). The residence time in the main compartments excluding intestine (Additional file 1: Table S11) is between 5 and 25 days. This comprises about 20% of the total iron (i.e. of ~2 mg per 25 g body [18]). The reminder is not readily accessible. The residence time of molecules here is ~200 days [18,19].

#### Iron turnover occurs at similar rate in intestine and skin, but assignment to iron loss vs. iron reflux is only indirectly estimable

Physiologically iron enters the body via duodenal and (less) small-intestinal absorption in a tightly controlled way. It leaves the body by desquamation, exfoliation of epithelial cells, by blood losses, and to a lesser extent via bile and urine [49]. The net amount leaving the murine body is, according to literature references, 2 to 5 times larger than in other animals and man [18,20,40,49-51]. A consequence of this higher excretion is that heavy iron-load is sometimes difficult to attain in mouse models.

Net iron losses cannot be measured by the tracer method as applied here. However, the fractional clearance rates (table 1) yield indirect information on iron fluxes through intestine and integument (Additional file 1: Table S8). Iron clearance of the epidermis integument is about 5% per day and that of the stomach-intestinal epithelium ca. 36% per day (calculated from table 1). From the fractional uptake from plasma one can calculate influx rates of ~1.7 μg per day into epidermis, and a sum of ~1.5 μg per day into intestine plus stomach (all for iron-adequate mouse, see Additional file 1: Table S8). These values are about 39% lower and higher in iron deficient and iron-load regimes, respectively. The data do not support a calculation of the rate of net iron loss through these compartments, because there may be a fraction that is recycled into plasma. The iron residence times for intestine are similar to the known exfoliation times of epithelium (3-5 days), which suggests that the main fraction goes into loss. For skin integument (iron residence about 40 days) such external information was not available.

#### Murine erythrocyte iron turnover has a random elimination component together with a lifespan-determined removal component

During one month after administration 60% of the tracer (40% in iron-loaded state) accumulates in the red blood cell compartment (figures 2, 3. The first quick uptake of ^59^Fe reflects passage through bone marrow and incorporation into hemoglobin at a steady rate. The uptake reaches a saturation phase which is clearly visible in the RBC curve of figures 2 to 3. This behaviour proves the existence of a reflux caused by a random component of erythrocyte catabolism independent of the cell age. Without reflux iron would be further incorporated even at a very low plasma radioactivity. The erythron cycle transports (Additional file 1: Table S8) 15;19;14 μg Fe/d into bone marrow in iron-deficient, -adequate, and -loaded animals, respectively, of which 12;17;12 μg Fe/d pass through the RBC compartment back via into RES into plasma. This turnover rate is quantitatively analogous to ~ 25 mg Fe/d per 70 kg in iron-adequate humans.

The life span of mouse erythrocytes has been studied in mathematical detail by Horký et al. [52]. They also formulated an age-independent linear elimination component acting simultaneously with a lifespan-determined senescence process. Our elimination rate (between 0.03 and 0.06 d^-1^) is somewhat higher than obtained in [52] (0.012d^-1^). However, these estimates are not very reliable, as they stem from an indirect deduction. This applies also to the size of the "readily accessible" iron pool in red blood cells (300 μg instead of the 568 μg calculated from the hemoglobin pool of the mouse, see Additional file 1: Table S9).

#### The spleen is a mixed indicator of erythropoiesis and RES activity

The murine spleen is an erythropoietic organ [53]. Therefore, one subcompartment of iron in the spleen is expected to behave similar to iron in the bone marrow. Figures 2, 3, and Additional file 1: Table S5 to S7 show a similarly quick uptake phase in both bone marrow and spleen. The ratio of tracer iron content between both organs after 12 h is about 50 to 60 in adequate and iron-rich mice, and 20 in iron-deficient animals. Thus, the quantitative contribution of the spleen to total murine erythropoiesis is not high. Furthermore, the iron-deficient spleen loses iron as quickly as the bone marrow, reflecting the rapid flow into "iron-deficient" erythropoiesis. In contrast to the bone marrow, the iron-adequate, and even more so the iron-loaded spleen retains ^59^Fe for long periods. This reflects a storage behaviour which is similar to that of RES cells in the liver and elsewhere. The spleen contains 5% and the liver 16% of the whole population of macrophages [54]. The RES system serves as scavenger to remove senescent erythrocytes together with their hemoglobin and colloidal iron from the circulation [8,55]. Part of this RES iron is rapidly recirculated into plasma, thereby completing the iron-recirculation back to the erythrocyte pool. Except in iron deficiency, another part of the RES iron is stored as ferritin or hemosiderin [44].

The quantitative contribution of both spleen compartments to whole body iron turnover is low. The spleen is therefore an indicator, but not the main quantitative locus of the total erythropoietic and macrophage activity. In iron-deficiency splenic iron clearance is very rapid (15% d^-1^, see table 1 and Additional file 2: Figure S1 and S2), while it is distinctly slower (down to 1.9% d^-1^) in iron loaded mice. This may reflect distinct differences in the role of the spleen depending on the state of iron-repletion. A precise quantitative partition of splenic iron fluxes into a RES-and an erythron-fraction would require separation of the cells.

#### Experimental design for characterizing the iron status and the dynamic turnover of the C57BL6 mouse strain

The C57BL6 mouse is a widely used strain for genetic modifications to address the regulatory networks of iron metabolism. Any such transgenic strain needs a characterization of its iron kinetics (examples in [56,57]). This includes a survey of static and dynamic characteristics of iron metabolism under the limitations set by thrifty experimental expense. The turnover model developed here permits to derive diagnostic requirements for healthy or diseased mice, after a steady-state has been established and maintained for the time of at least one red blood cell turnover. The following data should be scaled up to the total body level:

-plasma iron steady state, measured by transferrin level and its iron saturation

-liver and spleen total iron and non-heme iron (may be replaced by plasma ferritin as indicator)

-hemoglobin iron content, if possible red blood cell turnover (as indirect control of iron turnover

-hepcidin and erythropoietin levels

-plasma iron clearance rate constant (only possible with tracer injection and several measurements during the first 12 h)

-percentage uptake of tracer from duodenum (after a bolus dose into the stomach)

-organ content of tracer iron, blood-corrected, by several measurements between 12 and 72 h at least in liver, spleen, red blood cell compartment and bone marrow

-long-term rate of iron loss.

The biochemical parameters yield a survey of the static of iron metabolism and its steady-state level. Ferrokinetics yields the fluxes. This full programme can be reduced, if in a particular situation preliminary analysis of data and their comparison with the mathematical model indicate that certain features of the iron status are not changed or are negligible.

## Conclusion

The model of iron phenomenology in normal mice of different iron status is the basis for further development of a whole-body model, in which the homogenous compartments are being specified by internal kinetic models of different pertinent cell types. This will comprise the major pathways of iron uptake, iron secretion, biosynthesis of heme proteins, ferritin and hemosiderin storage (flux models) as well as the overarching regulatory structure (iron sensing, cellular regulation on the transcriptional and translational level, hormonal signalling between cell types) [34,35,58]. Our group intends to contribute to this goal by way of transgenic constructs targeted to iron-regulatory genes in specific cell types.

## Availability

The ferrokinetic models were submitted to the Biomodels - the public systems biology models repository - http://www.ebi.ac.uk/biomodels and are publicly available for download and use. The models are available under the unique IDs MODEL4152822384, MODEL4152801381 and MODEL4152760573 for Fe deficient, Fe adequate and Fe loaded, respectively.

## Methods

### Models, Data Processing and Parameter Estimation

#### Experimental design

The mathematical model presented here has been derived from our own experimental data [9]. In brief, male young adult mice (C57BL6 strain; 18-20 g) underwent a 5-week period (growth to 25 g) with a diet controlled for iron content (iron content of diet induction of deficiency: 6 mg/kg; for adequate supply: 180 mg/kg; for iron overload 25000 mg/kg). The experiment was started by intravenous administration of ionic radioactive tracer (Fe59 nitrate in complex with nitrolotriacetic acid) The single tracer dose contained about 0.285 μg per mouse, which is less than 2% of plasma iron and in the range of 0.01% of body iron. At certain intervals between 12 hours and 28 days animals (n = between 3 and 7) were sacrificed, blood was collected and their main organs were dissected, weighed, and their iron status (non-heme iron) was measured. Hematocrit and haemoglobin content of blood was measured, and aliquots were separated into plasma and red blood cell compartment. The weight of organs and tissue samples was measured and normalized to the whole body (25 g). The ambient Fe59 content of organs and blood compartments was measured by scintillation counting and also converted to a whole body value. The tissue contents of tracer were corrected by subtraction of the tracer in the residual blood, by a calculation scheme that was derived from parallel model experiments with Fe59-labeled erythrocytes as indicator [9].

All tracer data were corrected for the decay of radioactivity during the experiment by normalizing them with the help of the radioactivity of the injection solution measured at the same time.

Data concerning the total iron clearance rate could be taken from the literature [10], as they were obtained on control mice of the same strain under very similar dietary regimes.

#### General structure of iron metabolism in the mouse body

Mammalian organisms absorb iron from the intestinal tract, mainly via the duodenal epithelium, and loose it predominantly by exfoliation of intestinal epithelium, by desquamation of the skin, by occasional or repeated loss of blood, and to a lesser extent via excretion of a non-reabsorbed fraction of bile and urine. Duodenal absorption transfers iron into plasma where it is bound to transferrin. Transferrin-bound iron is distributed to peripheral tissues in accordance with their expression level of the transferrin receptor (TFR1). This stream into the periphery can be measured after injection of radioactive ^59^Fe into plasma as rate of appearance of the tracer in the periphery. This intake of iron into cells is balanced by an out stream back into plasma of similar strength, mediated by iron export protein ferroportin [11].

This general scheme of iron distribution into the periphery and its reflux into plasma is adequately represented by a "mammillary" compartment system with reversible flux (figure 1). Transferrin-bound iron equilibrates quickly (< 1 day) between plasma and extravascular fluid [2,12]. Together they are the central compartment. Other tissues form peripheral compartments. The erythropoetic compartment of bone marrow has a high expression level of the transporter TFR1 and rapidly integrates iron into hemoglobin [13,14]. Both fluxes as well as the filtering-out of senescent blood cells are irreversible. In contrast, the expression level of ferroportin in bone marrow is low. This allows us to model the iron pathway from plasma over bone marrow, erythrocyte pool, and RES (emphasized by thick arrows in figure 1) as a circular irreversible flux without reflux. A smaller flux from bone marrow into the Reticulo Endothelial System (RES) has to be included. It represents partly spleen erythropoiesis in mice and partly "ineffective erythropoiesis" [15].

Some of the peripheral compartments have two outlets, one leading back into the central compartment and the other one leaving the system by loss of cells or excretion. The clearance of radioactive tracer can be estimated in these cases, but not the partition between these two outlets. Loss of tracer from the body is difficult to measure, and the plasma curve is not sufficiently sensitive to small variants of back-flux from those tissues. For parameter estimation an *a priori *decision had to be made about which of the double outflows is quantitatively the more important. In the intestine and integument, with the exception of the duodenum, iron losses from the body are assumed to be the main route. This reflects the rapid exfoliation of intestinal epithelium (around 4 days), as well as the slower, but of larger volume, desquamation of skin and integumental adnexes. In the duodenum the ^59^Fe influx from plasma counters the physiological uptake of unlabelled iron from the lumen. In erythrocytes, liver, and kidney it was decided that the main pathway balancing iron uptake is reflux into plasma rather than loss out of the body.

The compartments in figure 1 represent organs and tissues. They are not kinetically homogenous, as every organ consists of cell types that may differ in their iron metabolism. Tracer content in such compartments and intercompartment fluxes represent therefore a weighted mean over different cell types. Mixture compartments of this type are liver, spleen and muscle.

#### Numerical Scales of Pools and Turnover Rates

The generic model of figure 1 has to be quantitatively specified. The assumed reference organism of all data in this paper is an adult mouse of 25 g body mass; all other references are transformed to this scale. Comparing relevant data from other species involves a scale factor of approximately 10 from mouse to rat and 2500 to 3000 from mouse to humans. Compartments are envisaged as iron aggregates ("pools") the iron content of which is a systemic variable, expressed in units of μg iron per animal. Fluxes into and out of a compartment are expressed in units of μg iron per animal per day.

The systemic structure of this model is specified by "content" data ("concentration" of iron in its various biochemical forms in the different compartments), and by "turnover" data, usually obtained from tracer studies. Both data sets must be scaled up to the whole organism. The model must have a mathematical structure that reflects statics and dynamics. Its parameters are to be estimated from the empirical data.

#### Mathematical structure of the model

The generic structure of the model is a set of balance equations describing time course and steady-state of the iron content of kinetically relevant pools:

(1) $$\frac{dC_{i}}{dt}=\sum_{j}v_{ij}-\sum_{j}v_{ji}+v_{io}-v_{oi}$$

where

C_i _( ≥ 0) - "pool size": iron content of the i-th compartment, i = 1...n (number of pools) - chosen scale is μg per body

v_ij _( ≥ 0) - rate of iron (in-) flux from compartment j to i (j = 1...n; j ≠ i) (μg per body per day)

v_ji _( ≥ 0) - rate of iron (out-)flux from compartment i to j (μg per body per day)

v_io _( ≥ 0) - rate of iron flux from outside the system into compartment i (μg per body per day); only influx into duodenum of "cold" iron has a value > 0

v_oi _( ≥ 0) - rate of iron flux from compartment i out of the system (μg per body per day; values > 0 only for intestine, stomach, integument).

#### Clearance mode of model description and derivation of motion equations

Each of the rates is a function of the status of some of the pool sizes and of kinetic parameters. One may introduce fractional clearance coefficients (letter k) by the following definitions:

(2) $$\begin{array}{l} k_{ij}\equiv \frac{v_{ij}}{C_{j}}\\ k_{oi}\equiv \frac{v_{oi}}{C_{i}} \end{array}$$

These coefficients describe the ambient tendency of a given flux to clear the pertinent source pool. They apply also to the dynamics of the tracer content. If fluxes and pool sizes do not change substantially during the experiment (steady-state of the bulk of "cold" iron fluxes and pools during the experiment by the tiny amount of tracer), the k's become constant and a system of ordinary linear differential equations with constant coefficients describes the motion of the tracer content. And a system of ordinary linear differential equations with constant coefficients (clearance rate constants) describes the ensuing relaxation (distribution of tracer governed by "cold" flux rates) into the steady-state as motion of the tracer content (x - being measured as specific radioactivity, i.e. counts per mass of compartment iron, normalized to initial tracer dose):

(3) $$\frac{{\text{d}}_{\text{xi}}}{{\text{d}}_{\text{t}}}=\sum_{j}k_{ij}*x_{j}-\sum_{j}k_{ji}*x_{i}-k_{oi}*x_{i}\;\;\;\;\left(i\;=\;1...n\right)$$

where a term k_io _* x_o _has been dropped because re-absorption of excreted radioactivity can be neglected, if the tracer has been applied to the plasma compartment. The k-values for non-existing fluxes (out of the system or non-reversible) are set to zero. The system of differential equations describing the tracer motion is written down in Additional file 3.

#### Residence time

Interpretation of systems dynamics is simplified by introduction of expected residence times of molecules of defined biochemical state within a compartment:

(4) $$\begin{array}{cc} \Theta \text{i}\equiv \frac{\text{1}}{\left(\sum_{\text{j}}{\text{k}}_{\text{ji}}\;+{\text{ k}}_{\text{oi}}\right)} & \text{(j}\;=\;1...\text{n};\;\text{j}\;\neq \;\text{i}) \end{array}$$

Ferrokinetic experiments on mice

The phenomenology of iron metabolism is measured by injection of radioactive iron into the central compartment. In mathematical terms this introduces the boundary conditions for the system (equation 3). Since the uptake of iron via the natural way (guts) is slow compared to the inner exchange rates, the radioactive tracer (by mass being less than 2% of total plasma iron) has to be introduced at time t = 0 intravenously in the form of transferrin-bound iron or of ionic salt that equilibrates quickly with transferrin-bound iron. Mixing in the central (= intravascular) compartment can be assumed to be quick, and is described as a "boundary condition" by x_plasma _(t = 0) = 100 (%). All other compartments have initial condition x(t = 0) = 0.

In iron-deficient mice the tracer content of the spleen dropped to zero after a short time and even reached apparent negative values. This is clearly an overcorrection for blood-related ^59^Fe, as the spleen contains a large extravascular blood pool that cannot be removed completely by perfusion. The true tracer content of the splenic tissue proper was obviously close to zero at those time points (see Additional file 1: Table S2). Therefore, we set these small negative values equal to zero. This manipulation did not appreciably change the fractional clearance parameter of the iron-deficient spleen.

#### Contribution of organs and tissues to whole body mass

An important scale factor is the fractional contribution of each organ or tissue to the mass of the whole body. Additional file 1: Table S4 provides data for organ weights of C57BLC mice litter mates from our previous publication [9]. We include the estimates of plasma and blood volume of the whole body corrected for the bias of peripheral venous measurement [16].

#### Iron content in tissues and organs and its interval of fluctuation

To scale up the specific iron concentration (f, expressed as μg iron per g wet organ) to the total contribution of an organ or tissue compartment we multiplied such data by the total weight w (in g) of the organ(s) per body, i.e. f * w. The standard deviation was calculated from the standard deviations h(f) and s(w) of the factors according to the formula 2.23 on p.7 of [17]:

(5) $${\text{ SQR (f}}^{\text{2}}{\text{*s}}^{\text{2}}+{\text{w}}^{\text{2}}{\text{*h}}^{\text{2}}+{\text{h}}^{\text{2}}{\text{*s}}^{\text{2}}\text{)}$$

which assumes that the measurements of organ mass and iron content are independent random variables.

#### Averaged tracer content in the intestine

As the precise organ weights of intestinal subsections, such as ileum/coecum/colon were not established, we replaced the tracer concentrations in Additional file 1: Table S1 to S3 by a mean ascribed to the intestine as a whole. The standard deviation of this derived quantity was calculated according to formula 5.

#### Normalization of tracer content of compartments to a common scale

It is technically difficult to inject exactly the same small fluid dose of radioactive iron-solution. Therefore, the ^59^Fe content of an organ at a given time point was expressed as the ^59^Fe concentration normalized according to the formula

(6) Sum of radioactivity in the body at time t=100%*exp{−0,005 t}

where t is measured in days.

This describes the long-term rate of iron loss from the murine body [18,19]. Bonnet et al. [20] give a somewhat lower rate of ~ 0.004 per day. The process of normalization smoothed the ups and downs of determined tracer contents. It indicates a loss of about 13% of the injected tracer over an experimental period of one month, which is in keeping with previous estimates (10-15%) [21].

#### Parameter estimation strategy

Model calculation and parameter estimation have recently undergone standardization and adaption to modern computing capacity [22-24]. We have applied a simplified simulation strategy, which was tailored to our needs. Incorporating actual data, the parameters of a model were point-estimated by optimization of fit-criteria. Such criteria measure the mathematical "distance", i.e. the squared difference between data (weighted according to precision) and a prediction obtained by simulation under a hypothesized parameter vector. The best-fitting parameter vector minimizes this distance. The "weighted non-linear least-squares"-method is an example for this strategy.

#### Fit Criteria

Data of fluctuating precision may be ranked by weight factors. We decided to use the inverse of the standard deviation of the measurements at a given time in the respective organs (called fval_chi_sqr in table 1). This led to better fits (estimated by inspection) than uniform weighting which overemphasizes high concentrations, or the use of the inverse of the variance which overemphasizes very low concentrations.

#### Quality of final fit

As an intuitive measure of the quality of the final fit we chose the root of mean of squared weighted deviation between prediction and the mean of measurements (table 1; "sq root of mean weighted squared dev").

#### Scatter interval of parameter estimates

Fluctuant data make the point estimates of parameters uncertain. We initially tried to estimate the related uncertainty by expanding a quadratic surface around the "optimal fit" and projecting its domain on the parameter scales. This is the usual maximum-likelihood method of estimation. However, since the real surface of the fit landscape was not approximately quadratic, and since the distribution of the data scatter did not harmonize with the assumptions of the theory, we transformed the scatter interval of measurements into a corresponding interval of realistic parameter estimates. This is a less assumption-laden strategy.

For this purpose we generated sets of new data by use of the Monte-Carlo resampling method. By randomization we obtained data that had the same mean and standard deviations as the actually measured data. The simulated data were subjected to the same parameter estimation and this yielded statistical information of the parameter uncertainty. We document the parameter set of the best fit together with an upper and lower bound obtained from a sextile-truncated sample of the empirically found parameter variation. In the case of a Gaussian distribution the interval thus defined would be twice the standard deviation.

#### Parameter identifiability

Some parameters or sets of parameters are not identifiable by measurement in a model of given design. For instance, if ferrokinetic measurements are available only for the time course of changes in plasma concentration of tracer, the total clearance rate can be adequately assessed, but not the distribution into the network of body compartments. In contrast, if the first measurement was obtained after most of the tracer has left the plasma compartment, the relative distribution to the various organs can be assessed, but the flux dynamics of this distribution cannot. Even if parameters seem to be identifiable in the Laplace domain [25], the error structure of the data will make the ensuing algebraic treatment ill-conditioned and will expand the range of parameter estimates. They become meaningless. We tackled this problem by studying the parameter space using the alternating conditional expectation algorithm (ACE) [26,27].

The essence of this strategy is to explore the total domain with all parameter values that support an acceptable fit, i.e. the criterion value of this fit is sufficiently close to the optimum. This is done by running a large number of parameter estimations from a large range of starting points. The result is a set of sometimes widely differing parameter vectors that yield very similar optimal values of the fit criterion. Interdependent and hence non-identifiable parameter combinations can be addressed by the information furnished by the ACE method. On the basis of this analysis one or more suitable parameters are chosen to be fixed or held in definite proportion to each other so that the other parameters became identifiable. The choice of suitable parameters for this reduction of parameter redundancy and of their fixed values is not always easy and requires a theoretical understanding of the dynamic structure of the model. Maiwald et al. [28] have developed software ("Potter's wheel") the tools of which support this computer-time-intensive study very effectively. Specific assumptions made to remove strong parameter intercorrelation will be mentioned explicitly below.

#### Calculation of absolute flux rates

The tracer data alone can be used to estimate fractional clearance parameters (per day) of flux out of plasma (k_i_plasma_). To calculate flux rates (μg per day per mouse) one needs the iron content of plasma plus extracellular fluid (C_plasma/ECF_):

(7) $${\text{v}}_{\text{i\_plasma}}={\text{k}}_{\text{i\_plasma}}*{\text{C}}_{\text{plasma/ECF}}$$

The iron pool of the plasma plus ECF is calculable from the iron concentration and the plasma volume plus the accessible volume of ECF

#### Estimation of peripheral pool size

The iron content of body organs can be measured by chemical methods, mainly as hemoglobin, myoglobin, non-heme iron. Cellular heme iron content as oxidoreductases and other proteins is less important quantitatively. Such direct measurements may be contrasted with tracer-accessible iron pool sizes. When in the steady-state influx and outflux are equal, the following equation relates the pool size C_i _of a peripheral compartment to that of plasma

(8) $${\text{k}}_{\text{i\_plasma}}*{\text{C}}_{\text{plasma/ECF}}={\text{k}}_{\text{plasma\_i}}*{\text{C}}_{\text{i}}$$

If the data support estimates of the two rate constants (k_i_plasma _defining the early phase and k_plasma_i _characterizing the late phase of tracer distribution) and of the plasma/ECF iron content, then the size of the "accessible" pool C_i _may be inferred.

## Authors' contributions

JGR conceived the study and wrote the manuscript. TJSL implemented the programs, prepared the figures, tables and helped writing the manuscript. TL helped organizing the bibliography and the quantitative information, MVS, MWH, MUM and KS contributed with their expertise in the iron field, interpretation of the results and writing the manuscript. All authors read and approved the final manuscript

## Supplementary Material

Additional file 1 **This file comprises 13 tables with all the quantitative information used and calculated in this study**. Click here for file

Additional file 2 **This file contains 4 supplementary figures describing graphically our model layout and some information mentioned in the manuscript text**. Click here for file

Additional file 3 **This has file has the differential equations that compose our mathematical model**. Click here for file

## References

[B1] Hentze MW Muckenthaler MU Andrews NC Balancing acts: molecular control of mammalian iron metabolism Cell 2004 117 285 297 10.1016/S0092-8674(04)00343-5 15109490

[B2] Finch CA Deubelbeiss K Cook JD Eschbach JW Harker LA Funk DD Marsaglia G Hillman RS Slichter S Adamson JW Ferrokinetics in man Medicine (Baltimore) 1970 49 17 53 4908580 10.1097/00005792-197001000-00002

[B3] McCance RAWEM Absorption and excretion of iron Lancet 1937 230 5 10.1016/S0140-6736(00)91144-9

[B4] Vacha J Znojil V Hola J Dungel J The Internal Iron Kinetics in Mice ACTA VET BRNO 1982 51 3 22 10.2754/avb198251010003

[B5] Nathanson MH Muir A McLaren GD Iron absorption in normal and iron-deficient beagle dogs: mucosal iron kinetics Am J Physiol 1985 249 G439 448 4050995 10.1152/ajpgi.1985.249.4.G439

[B6] Berzuini C Franzone PC Stefanelli M Viganotti C Iron kinetics: modelling and parameter estimation in normal and anemic states Comput Biomed Res 1978 11 209 227 10.1016/0010-4809(78)90008-3 679654

[B7] Pollycove M Mortimer R The quantitative determination of iron kinetics and hemoglobin synthesis in human subjects J Clin Invest 1961 40 753 782 10.1172/JCI104310 13736888 PMC290786

[B8] Stefanelli M Bentley DP Cavill I Roeser HP Quantitation of reticuloendothelial iron kinetics in humans Am J Physiol 1984 247 R842 849 6496771 10.1152/ajpregu.1984.247.5.R842

[B9] Schumann K Szegner B Kohler B Pfaffl MW Ettle T A method to assess 59Fe in residual tissue blood content in mice and its use to correct 59Fe-distribution kinetics accordingly Toxicology 2007 241 19 32 10.1016/j.tox.2007.08.082 17868968

[B10] Trinder D Olynyk JK Sly WS Morgan EH Iron uptake from plasma transferrin by the duodenum is impaired in the Hfe knockout mouse Proc Natl Acad Sci USA 2002 99 5622 5626 10.1073/pnas.082112299 11943867 PMC122820

[B11] Ganz T Cellular iron: ferroportin is the only way out Cell Metab 2005 1 155 157 10.1016/j.cmet.2005.02.005 16054057

[B12] Morgan EH Worwood AJaM Transferrin and transferrin iron Iron in Biochemistry and Medicine 1974 London and New York: Academic Press 29 71

[B13] Allen DW Jandl JH Kinetics of intracellular iron in rabbit reticulocytes Blood 1960 15 71 81 13792723

[B14] Noyes WD Hosain F Finch CA Incorporation of Radioiron into Marrow Heme J Lab Clin Med 1964 64 574 580 14233146

[B15] Ricketts C Jacobs A Cavill I Ferrokinetics and erythropoiesis in man: the measurement of effective erythropoiesis, ineffective erythropoiesis and red cell lifespan using 59Fe Br J Haematol 1975 31 65 75 10.1111/j.1365-2141.1975.tb00833.x 1212437

[B16] Vacha J Blood volume in inbred strain BALB/c, CBA/J and C57BL/10 mice determined by means of 59Fe-labelled red cells and 59Fe bound to transferrin Physiol Bohemoslov 1975 24 413 419 127187

[B17] Blumenfeld D Operations Research Calculations Handbook 2001 1 Boca Raton, London, New York, Washington: CRC Press

[B18] Chappelle E Gabrio BW Stevens AR Jr Finch CA Regulation of body iron content through excretion in the mouse Am J Physiol 1955 182 390 392 13258820 10.1152/ajplegacy.1955.182.2.390

[B19] Stevens AR Jr White PL Hegsted DM Finch CA Iron excretion in the mouse J Biol Chem 1953 203 161 165 13069499

[B20] Bonnet JD Orvis AL Hagedorn AB Owen CA Jr Rate of loss of radioiron from mouse and man Am J Physiol 1960 198 784 786 13802571 10.1152/ajplegacy.1960.198.4.784

[B21] Lebeau A Frank J Biesalski HK Weiss G Srai SK Simpson RJ McKie AT Bahram S Gilfillan S Schumann K Long-term sequelae of HFE deletion in C57BL/6 × 129/O1a mice, an animal model for hereditary haemochromatosis Eur J Clin Invest 2002 32 603 612 10.1046/j.1365-2362.2002.01026.x 12190960

[B22] Mendes P Kell D Non-linear optimization of biochemical pathways: applications to metabolic engineering and parameter estimation Bioinformatics 1998 14 869 883 10.1093/bioinformatics/14.10.869 9927716

[B23] Moles CG Mendes P Banga JR Parameter estimation in biochemical pathways: a comparison of global optimization methods Genome Res 2003 13 2467 2474 10.1101/gr.1262503 14559783 PMC403766

[B24] Zi Z Klipp E SBML-PET: a Systems Biology Markup Language-based parameter estimation tool Bioinformatics 2006 22 2704 2705 10.1093/bioinformatics/btl443 16926221

[B25] Nathanson MH Saidel GM Mclaren GD Analysis of Iron Kinetics - Identifiability, Experiment Design, and Deterministic Interpretations of a Stochastic-Model Mathematical Biosciences 1984 68 1 21 10.1016/0025-5564(84)90072-5

[B26] Breiman L Friedman JH Estimating Optimal Transformations for Multiple-Regression and Correlation - Rejoinder Journal of the American Statistical Association 1985 80 614 619 10.2307/2288477

[B27] Hengl S Kreutz C Timmer J Maiwald T Data-based identifiability analysis of non-linear dynamical models Bioinformatics 2007 23 2612 2618 10.1093/bioinformatics/btm382 17660526

[B28] Maiwald T Timmer J Dynamical modeling and multi-experiment fitting with PottersWheel Bioinformatics 2008 24 2037 2043 10.1093/bioinformatics/btn350 18614583 PMC2530888

[B29] Cavill I Ricketts C Napier JA Jacobs A Trevett D Bishop RD The measurement of 59Fe clearance from the plasma Scand J Haematol 1976 17 160 166 10.1111/j.1600-0609.1976.tb01171.x 968447

[B30] Cavill I Ricketts C Erythropoiesis and iron kinetics Br J Haematol 1978 38 433 437 10.1111/j.1365-2141.1978.tb01068.x 348226

[B31] Cavill I Ricketts C Jacobs A Radioiron and erythropoiesis: methods, interpretation and clinical application Clin Haematol 1977 6 583 599 912956

[B32] Finch C Regulators of iron balance in humans Blood 1994 84 1697 1702 8080980

[B33] Glass RD Doyle D On the measurement of protein turnover in animal cells J Biol Chem 1972 247 5234 5242 4626918

[B34] Andrews NC Disorders of iron metabolism N Engl J Med 1999 341 1986 1995 10.1056/NEJM199912233412607 10607817

[B35] Andrews NC Schmidt PJ Iron homeostasis Annu Rev Physiol 2007 69 69 85 10.1146/annurev.physiol.69.031905.164337 17014365

[B36] Bahram S Gilfillan S Kuhn LC Moret R Schulze JB Lebeau A Schumann K Experimental hemochromatosis due to MHC class I HFE deficiency: immune status and iron metabolism Proc Natl Acad Sci USA 1999 96 13312 13317 10.1073/pnas.96.23.13312 10557317 PMC23944

[B37] Cavill I The preparation of 59 Fe-labelled transferrin for ferrokinetic studies J Clin Pathol 1971 24 472 474 10.1136/jcp.24.5.472 5571840 PMC477030

[B38] Cavill I Ricketts C JAaW M Human iron kinetics Iron in biochemistry and medicine 1974 London: Academic Press 769

[B39] Fillet G Cook JD Finch CA Storage iron kinetics. VII. A biologic model for reticuloendothelial iron transport J Clin Invest 1974 53 1527 1533 10.1172/JCI107703 4830220 PMC302648

[B40] Kaufman RM Pollack S Anderson P Crosby WH Effect of Hemolysis on Excretion and Accumulation of Iron in the Rat Am J Physiol 1964 207 1041 1043 14237446 10.1152/ajplegacy.1964.207.5.1041

[B41] Kaufman RM Pollack S Crosby WH Iron-deficient diet: effects in rats and humans Blood 1966 28 726 737 5922678

[B42] Wick MPW Lehmann P Clinical Aspects and Laboratory Iron Metabolism 2003

[B43] Donovan A Brownlie A Zhou Y Shepard J Pratt SJ Moynihan J Paw BH Drejer A Barut B Zapata A Positional cloning of zebrafish ferroportin1 identifies a conserved vertebrate iron exporter Nature 2000 403 776 781 10.1038/35001596 10693807

[B44] Harrison PM Arosio P The ferritins: molecular properties, iron storage function and cellular regulation Biochim Biophys Acta 1996 1275 161 203 10.1016/0005-2728(96)00022-9 8695634

[B45] Haskins D Stevens AR Jr Finch S Finch CA Iron metabolism; iron stores in man as measured by phlebotomy J Clin Invest 1952 31 543 547 10.1172/JCI102639 14938431 PMC436450

[B46] Cook JD Hershko C Finch CA Storage iron kinetics. IV. Cellular distribution of ferritin iron stores in rat liver Proc Soc Exp Biol Med 1974 145 1378 1381 4827748 10.3181/00379727-145-38017

[B47] Hershko C Cook JD Finch CA Storage iron kinetics. VI. The effect of inflammation on iron exchange in the rat Br J Haematol 1974 28 67 75 10.1111/j.1365-2141.1974.tb06640.x 4415906

[B48] Van Wyk CP Linder-Horowitz M Munro HN Effect of iron loading on non-heme iron compounds in different liver cell populations J Biol Chem 1971 246 1025 1031 5543667

[B49] Hahn PF WFB RA Hettig M Kamen D Whipple GH Radioactive iron and its excretion in the urine, bile and feces Journal of Experimental Medicine 1939 70 8 10.1084/jem.70.5.443 PMC2133804 19870921

[B50] Dubach R Moore CV Callender S Studies in iron transportation and metabolism. IX. The excretion of iron as measured by the isotope technique J Lab Clin Med 1955 45 599 615 14368024

[B51] Forrester RH Conrad ME Jr Crosby WH Measurement of total body iron in animals using whole-body liquid scintillation detectors Proc Soc Exp Biol Med 1962 111 115 119 13958785 10.3181/00379727-111-27718

[B52] Horky J Vacha J Znojil V Comparison of life span of erythrocytes in some inbred strains of mouse using 14C-labelled glycine Physiol Bohemoslov 1978 27 209 217 150611

[B53] Brodsky I Dennis LH Kahn SB Brady LW Normal mouse erythropoiesis. I. The role of the spleen in mouse erythropoiesis Cancer Res 1966 26 198 201 5903171

[B54] Lee SH Starkey PM Gordon S Quantitative analysis of total macrophage content in adult mouse tissues. Immunochemical studies with monoclonal antibody F4/80 J Exp Med 1985 161 475 489 10.1084/jem.161.3.475 3973536 PMC2187577

[B55] Knutson M Wessling-Resnick M Iron metabolism in the reticuloendothelial system Crit Rev Biochem Mol Biol 2003 38 61 88 10.1080/713609210 12641343

[B56] Muckenthaler M Roy CN Custodio AO Minana B deGraaf J Montross LK Andrews NC Hentze MW Regulatory defects in liver and intestine implicate abnormal hepcidin and Cybrd1 expression in mouse hemochromatosis Nat Genet 2003 34 102 107 10.1038/ng1152 12704390

[B57] Vujic Spasic M Kiss J Herrmann T Kessler R Stolte J Galy B Rathkolb B Wolf E Stremmel W Hentze MW Muckenthaler MU Physiologic systemic iron metabolism in mice deficient for duodenal Hfe Blood 2007 109 4511 4517 10.1182/blood-2006-07-036186 17264297

[B58] Nemeth E Ganz T Regulation of iron metabolism by hepcidin Annu Rev Nutr 2006 26 323 342 10.1146/annurev.nutr.26.061505.111303 16848710

[B59] Noyes WD Bothwell TH Finch CA The role of the reticulo-endothelial cell in iron metabolism Br J Haematol 1960 6 43 55 10.1111/j.1365-2141.1960.tb06216.x 14427868

