# Supplementary material for: Systems analysis of iron metabolism: the network of iron pools and fluxes
Source: BMC Syst Biol. 2010 Aug 13;4:112. doi: 10.1186/1752-0509-4-112 (PMC2942822; doi:10.1186/1752-0509-4-112)
Supplement: Additional file 1 — This file comprises 13 tables with all the quantitative information used and calculated in this study. [file 1752-0509-4-112-S1.PDF]

Table S1: Iron-adequate Diet in healthy Mice - Tracer Iron Content of Body Organs after intravenous Injection

Organ / Time	0.5 Day	1 Day	4 Days	7 Days	14 Days	28 Days
**Duodenum**	0.555 ± 0.222	0.349 ± 0.148	0.207 ± 0.055	0.052 ± 0.002	0.085 ± 0.032	0.047 ± 0.019
**Liver**	0.849 ± 0.249	0.706 ± 0.317	0.664 ± 0.105	0.547 ± 0.076	0.432 ± 0.199	0.258 ± 0.075
**Spleen**	0.831 ± 0.196	1.153 ± 0.471	0.619 ± 0.517	0.582 ± 0.452	0.398 ± 0.111	0.438 ± 0.114
**Bones**	1.937 ± 0.587	0.720 ± 0.247	0.381 ± 0.085	0.282 ± 0.083	0.251 ± 0.163	0.124 ± 0.031
**Heart**	0.520 ± 0.090	0.260 ± 0.040	0.400 ± 0.170	0.400 ± 0.330	0.370 ± 0.260	0.230 ± 0.080
**Kidneys**	0.500 ± 0.200	0.310 ± 0.160	0.350 ± 0.120	0.170 ± 0.050	0.170 ± 0.150	0.180 ± 0.170
**Lungs**	0.780 ± 0.480	1.170 ± 1.160	0.490 ± 0.110	0.330 ± 0.420	0.330 ± 0.120	0.300 ± 0.120
**Stomach**	0.240 ± 0.160	0.180 ± 0.100	0.100 ± 0.030	0.040 ± 0.020	0.060 ± 0.030	0.050 ± 0.030
**Integument**	0.150 ± 0.190	0.100 ± 0.040	0.140 ± 0.040	0.150 ± 0.190	0.180 ± 0.070	0.070 ± 0.060
**Fat**	0.040 ± 0.020	0.060 ± 0.030	0.050 ± 0.030	0.110 ± 0.100	0.050 ± 0.050	0.010 ± 0.010
**Muscle**	0.060 ± 0.030	0.030 ± 0.010	0.030 ± 0.010	0.080 ± 0.100	0.010 ± 0.010	0.020 ± 0.010
**Brain**	0.030 ± 0.010	0.020 ± 0.010	0.030 ± 0.010	0.020 ± 0.100	0.030 ± 0.020	0.020 ± 0.000
**Testes**	0.080 ± 0.020	0.080 ± 0.010	0.090 ± 0.020	0.070 ± 0.040	0.080 ± 0.030	0.040 ± 0.010
**Plasma**	0.087 ± 0.048	0.015 ± 0.007	0.011 ± 0.007	0.005 ± 0.001	0.013 ± 0.001	0.016 ± 0.011
**Blood**	0.860 ± 0.380	1.730 ± 0.640	2.220 ± 0.600	1.780 ± 0.490	1.760 ± 0.380	1.880 ± 0.240
**Intestine**	0.370 ± 0.220	0.300 ± 0.130	0.160 ± 0.100	0.050 ± 0.020	0.040 ± 0.070	0.030 ± 0.080
**Ileum**	0.490 ± 0.280	0.350 ± 0.120	0.140 ± 0.040	0.040 ± 0.010	0.040 ± 0.120	0.030 ± 0.100
**Caecum**	0.310 ± 0.170	0.320 ± 0.140	0.230 ± 0.170	0.040 ± 0.020	0.030 ± 0.010	0.020 ± 0.100
**Colon**	0.300 ± 0.190	0.220 ± 0.130	0.120 ± 0.040	0.060 ± 0.030	0.040 ± 0.020	0.040 ± 0.020

Data (nmoles Fe^{59} per g wet weight after an intravenous injection of tracer) are from tables 3 and 4 of [9]. They apply to organ content after correction for residual blood content not removed by perfusion.

The intestinal content was calculated as mean ± standard deviation (rounded to 2 digits) from the values of ileum/caecum/colon (standard deviation of intestine: root of the mean of the three variances).

For plasma and whole blood data were taken from Figure 2 of [9]. The tracer content in the original data was normalized to the iron concentration in the initial dose of administered radioactivity.

Table S2: Iron-deficient Diet in healthy Mice - Tracer Iron Content of Body Organs after intravenous Injection

Organ / Time	0.5 DAY	1 DAY	4 DAYS	7 DAYS	14 DAYS	28 DAYS
**Duodenum**	0.282 ± 0.130	0.133 ± 0.036	0.167 ± 0.046	0.069 ± 0.028	0.093 ± 0.064	0.081 ± 0.030
**Liver**	0.559 ± 0.214	0.494 ± 0.276	0.539 ± 0.184	0.259 ± 0.056	0.230 ± 0.110	0.108 ± 0.027
**Spleen**	1.279 ± 0.346	0.431 ± 0.303	0.085 ± 0.143	**-0.064** ± 0.105	**-0.151** ± 0.165	**-0.062** ± 0.077
**Bones**	1.053 ± 0.265	0.221 ± 0.101	0.165 ± 0.062	0.096 ± 0.028	0.041 ± 0.026	0.064 ± 0.012
**Heart**	0.330 ± 0.120	0.270 ± 0.180	0.370 ± 0.090	0.330 ± 0.230	0.260 ± 0.090	0.410 ± 0.140
**Kidneys**	0.360 ± 0.190	0.290 ± 0.150	0.360 ± 0.090	0.150 ± 0.090	0.150 ± 0.160	0.190 ± 0.050
**Lungs**	0.890 ± 0.820	2.600 ± 0.750	1.220 ± 0.950	0.280 ± 0.140	0.480 ± 0.240	0.160 ± 0.090
**Stomach**	0.210 ± 0.120	0.120 ± 0.080	0.150 ± 0.050	0.100 ± 0.070	0.080 ± 0.040	0.090 ± 0.030
**Integument**	0.130 ± 0.040	0.100 ± 0.040	0.170 ± 0.090	0.110 ± 0.070	0.090 ± 0.050	0.190 ± 0.050
**Fat**	0.040 ± 0.010	0.060 ± 0.040	0.050 ± 0.030	0.060 ± 0.060	0.040 ± 0.030	0.030 ± 0.010
**Muscle**	0.030 ± 0.010	0.040 ± 0.040	0.030 ± 0.020	0.030 ± 0.020	0.020 ± 0.020	0.050 ± 0.010
**Brain**	0.020 ± 0.001	0.020 ± 0.010	0.030 ± 0.010	0.040 ± 0.020	0.040 ± 0.010	0.040 ± 0.010
**Testes**	0.070 ± 0.010	0.050 ± 0.020	0.070 ± 0.020	0.080 ± 0.030	0.100 ± 0.060	0.070 ± 0.020
**Plasma**	0.080 ± 0.046	0.041 ± 0.030	0.017 ± 0.004	0.017 ± 0.019	0.012 ± 0.008	0.017 ± 0.015
**Blood**	0.880 ± 0.160	2.000 ± 0.890	2.640 ± 0.500	2.720 ± 0.780	2.920 ± 1.040	2.760 ± 0.320
**Intestine**	0.337 ± 0.167	0.223 ± 0.104	0.170 ± 0.072	0.093 ± 0.098	0.053 ± 0.027	0.037 ± 0.060
**Ileum**	0.360 ± 0.180	0.270 ± 0.050	0.140 ± 0.090	0.060 ± 0.060	0.050 ± 0.020	0.040 ± 0.020
**Caecum**	0.360 ± 0.160	0.260 ± 0.170	0.210 ± 0.070	0.090 ± 0.050	0.050 ± 0.030	0.020 ± 0.100

Data (nmoles Fe^{59} per g wet weight after an intravenous injection of tracer) are from tables 3 and 4 of [9]. They apply to organ content after correction for residual blood content not removed by perfusion. The intestinal content was calculated as mean ± standard deviation (rounded to 2 digits) from the values of ileum/caecum/colon (standard deviation of intestine: root of the mean of the three variances). For plasma and whole blood data were taken from Figure 2 of [9]. The tracer content in the original data was normalized to the iron concentration in the initial dose of administered radioactivity. Three values (spleen, iron-deficient at 7, 14, 28 days) were slightly lower than zero within a standard deviation interval covering the zero value. This is due to over-correction of blood-content (see methods section "Organ mass of the whole body") in the original publication.

Table S3: Iron-loaded Diet in healthy Mice - Tracer Iron Content of Body Organs after intravenous Injection

Organ / Time	0.5 Day	1 Day	4 Days	7 Days	14 Days	28 Days
**Duodenum**	0.266 ± 0.140	0.469 ± 0.103	0.291 ± 0.221	0.074 ± 0.038	0.105 ± 0.092	0.103 ± 0.090
**Liver**	0.769 ± 0.257	2.332 ± 0.406	1.505 ± 0.763	0.840 ± 0.276	0.895 ± 0.586	0.898 ± 0.238
**Spleen**	0.526 ± 0.352	0.986 ± 0.511	0.633 ± 0.007	0.709 ± 0.344	0.632 ± 0.102	0.928 ± 0.217
**Bones**	1.062 ± 0.368	0.686 ± 0.203	0.234 ± 0.047	0.191 ± 0.066	0.304 ± 0.094	0.176 ± 0.034
**Heart**	0.670 ± 0.200	0.810 ± 0.280	0.640 ± 0.170	0.740 ± 0.430	0.300 ± 0.150	0.270 ± 0.180
**Kidneys**	0.750 ± 0.530	1.700 ± 0.460	1.000 ± 0.900	0.470 ± 0.260	0.410 ± 0.300	0.350 ± 0.200
**Lungs**	1.410 ± 1.110	1.340 ± 0.320	1.460 ± 1.350	3.050 ± 0.800	1.260 ± 1.250	0.800 ± 0.340
**Stomach**	0.230 ± 0.240	0.620 ± 0.260	0.220 ± 0.160	0.140 ± 0.120	0.110 ± 0.100	0.120 ± 0.080
**Integument**	0.100 ± 0.050	0.200 ± 0.040	0.080 ± 0.060	0.100 ± 0.050	0.090 ± 0.040	0.090 ± 0.030
**Fat**	0.040 ± 0.020	0.090 ± 0.040	0.060 ± 0.040	0.070 ± 0.030	0.070 ± 0.010	0.020 ± 0.020
**Muscle**	0.040 ± 0.020	0.080 ± 0.030	0.040 ± 0.020	0.040 ± 0.010	0.040 ± 0.020	0.020 ± 0.010
**Brain**	0.010 ± 0.010	0.020 ± 0.001	0.010 ± 0.010	0.020 ± 0.010	0.030 ± 0.010	0.020 ± 0.001
**Testes**	0.100 ± 0.120	0.070 ± 0.020	0.040 ± 0.010	0.060 ± 0.010	0.070 ± 0.030	0.050 ± 0.010
**Plasma**	0.085 ± 0.024	0.026 ± 0.013	0.006 ± 0.002	0.004 ± 0.001	0.007 ± 0.005	0.013 ± 0.005
**Blood**	0.240 ± 0.052	0.520 ± 0.164	0.800 ± 0.080	1.200 ± 0.180	1.840 ± 0.400	1.280 ± 0.256
**Intestine**	0.233 ± 0.145	0.387 ± 0.137	0.167 ± 0.131	0.083 ± 0.054	0.077 ± 0.034	0.063 ± 0.044
**Ileum**	0.250 ± 0.160	0.360 ± 0.140	0.150 ± 0.110	0.060 ± 0.050	0.040 ± 0.010	0.040 ± 0.010
**Caecum**	0.220 ± 0.110	0.400 ± 0.140	0.170 ± 0.130	0.080 ± 0.050	0.080 ± 0.030	0.060 ± 0.030
**Colon**	0.230 ± 0.160	0.400 ± 0.130	0.180 ± 0.150	0.110 ± 0.060	0.110 ± 0.050	0.090 ± 0.070

Data (nmoles Fe^{59} per g wet weight after an intravenous injection of tracer) are from tables 3 and 4 of [9]. They apply to organ content after correction for residual blood content not removed by perfusion. The intestinal content was calculated as mean ± standard deviation (rounded to 2 digits) from the values of ileum/caecum/colon (standard deviation of intestine: root of the mean of the three variances).
For plasma and whole blood data were taken from Figure 2 of [9].
The tracer content in the original data was normalized to the iron concentration in the initial dose of administered radioactivity.

Table S4: Organ weights of C57BL6 Mice

Organ weight in g per animal (ca 25 g)		Standard deviation	% of body weight
Liver*	1.22	0.10	4.75
Spleen	0.07	0.01	0.27
Bones	1.80	0.25	7.01
Heart	0.14	0.02	0.55
Kidneys	0.38	0.05	1.48
Lungs	0.13	0.05	0.51
Stomach	0.18	0.04	0.70
Intestine	1.22	0.19	4.75
Duodenum	0.04	0.01	0.16
Integument (fur)	3.97	0.73	15.50
Fat	0.31	0.07	1.21
Muscle	13.42	1.21	52.30
Brain	0.47	0.01	1.83
Testicles	0.24	0.07	0.94
Plasma	1.36	0.03	5.30
Blood cells	0.71	0.02	2.77
Whole blood**	2.07	0.04	8.07
Bone marrow***	0.21	0.03	0.84

* Liver weight per mouse. Data from [9]
** Barbee et al. [60] give 2.3 ml / 25 g mouse
*** Mass of bone marrow in mouse (body weight 23 g) calculated from Lee et al.([54] p.484) is 192 mg, here scaled up to a mouse body of 25 g.

The values were taken from [9]. Blood values were scaled to the whole body hematocrit according to [16].
It is being assumed that the organ weights do not considerably change between different dietary regimes in a healthy mouse.

Table S5: Organ Fe59 Contents scaled to the whole mouse body (iron-adequate diet)

Organ/Time	0.5 Day	1 Day	4 Days	7 Days	14 Days	28 Days
Duodenum	0.255 ± 0.123	0.188 ± 0.095	0.107 ± 0.040	0.029 ± 0.007	0.055 ± 0.025	0.031 ± 0.015
Liver	11.903 ± 3.636	11.626 ± 5.323	10.432 ± 1.863	9.406 ± 1.521	8.536 ± 4.007	5.256 ± 1.592
Spleen	0.669 ± 0.186	1.089 ± 0.476	0.558 ± 0.477	0.574 ± 0.458	0.451 ± 0.143	0.512 ± 0.153
Bones	40.069 ± 13.463	17.493 ± 6.528	8.832 ± 2.337	7.155 ± 2.347	7.317 ± 4.904	3.727 ± 1.074
Heart	0.837 ± 0.189	0.491 ± 0.104	0.721 ± 0.326	0.789 ± 0.667	0.839 ± 0.607	0.538 ± 0.204
Kidneys	2.184 ± 0.927	1.590 ± 0.854	1.713 ± 0.634	0.911 ± 0.295	1.046 ± 0.941	1.142 ± 1.098
Lungs	1.165 ± 0.890	2.053 ± 2.319	0.820 ± 0.372	0.605 ± 0.857	0.695 ± 0.380	0.651 ± 0.375
Stomach	0.496 ± 0.357	0.437 ± 0.267	0.232 ± 0.088	0.101 ± 0.057	0.175 ± 0.098	0.150 ± 0.098
Intestine	5.141 ± 3.204	4.885 ± 2.300	2.566 ± 1.693	0.802 ± 0.396	0.725 ± 1.414	0.611 ± 1.703
Integument	6.844 ± 8.903	5.358 ± 2.392	7.158 ± 2.461	8.394 ± 10.920	11.574 ± 5.047	4.640 ± 4.133
Fat	0.143 ± 0.080	0.251 ± 0.141	0.200 ± 0.131	0.481 ± 0.461	0.251 ± 0.264	0.052 ± 0.054
Muscle	9.254 ± 4.720	5.434 ± 1.884	5.185 ± 1.797	15.133 ± 19.042	2.174 ± 2.191	4.482 ± 2.286
Brain	0.162 ± 0.054	0.127 ± 0.064	0.182 ± 0.061	0.132 ± 0.663	0.228 ± 0.152	0.157 ± 0.003
Testes	0.221 ± 0.086	0.259 ± 0.083	0.278 ± 0.104	0.237 ± 0.157	0.311 ± 0.152	0.160 ± 0.063
Plasma	1.358 ± 0.749	0.266 ± 0.123	0.191 ± 0.128	0.092 ± 0.019	0.293 ± 0.023	0.367 ± 0.238
Blood-Plasma	19.051 ± 9.058	47.953 ± 17.864	58.846 ± 15.996	51.719 ± 14.297	58.570 ± 12.757	64.458 ± 8.366

The organ content at a given time point was obtained by multiplying the measured specific content (Additional file 1: Table S1, [nmoles Fe^{59} per g wet weight]) by the organ weight per body (Additional file 1: Table S4, [g per mouse]). The radioactivities are scaled to give an initial plasma value of 100. For calculation of standard deviations, see methods section “Iron content in body organs and its range of fluctuation”.

The resulting dimensionless columns for each time point (measured in days) were recalculated proportionally to a tracer sum of the whole body of 100% * exp (-0.005 * t). This takes into account that the total iron content of the body is diminished at a rate of 0.5% per day [19].

Table S6: Organ Fe59 Contents scaled to the whole mouse body (iron-deficient diet)

Organ/Time	0.5 Day	1 Day	4 Days	7 Days	14 Days	28 Days
Duodenum	0.181 ± 0.097	0.076 ± 0.029	0.081 ± 0.030	0.037 ± 0.018	0.047 ± 0.036	0.037 ± 0.017
Liver	10.941 ± 4.297	8.666 ± 4.910	7.934 ± 2.794	4.194 ± 0.973	3.582 ± 1.744	1.501 ± 0.396
Spleen	1.436 ± 0.443	0.434 ± 0.314	0.072 ± 0.122	0.000 ± 0.150	0.000 ± 0.150	0.000 ± 0.150
Bones	30.695 ± 8.824	5.720 ± 2.756	3.583 ± 1.448	2.293 ± 0.747	0.942 ± 0.617	1.312 ± 0.308
Heart	0.741 ± 0.292	0.544 ± 0.374	0.625 ± 0.178	0.613 ± 0.440	0.465 ± 0.176	0.654 ± 0.244
Kidneys	2.195 ± 1.203	1.585 ± 0.853	1.650 ± 0.469	0.756 ± 0.468	0.728 ± 0.789	0.823 ± 0.244
Lungs	1.856 ± 1.966	4.860 ± 2.398	1.914 ± 1.758	0.483 ± 0.319	0.797 ± 0.525	0.237 ± 0.169
Stomach	0.606 ± 0.380	0.311 ± 0.223	0.326 ± 0.133	0.239 ± 0.179	0.184 ± 0.103	0.185 ± 0.075
Intestine	6.589 ± 3.462	3.918 ± 1.941	2.502 ± 1.139	1.511 ± 1.617	0.831 ± 0.446	0.510 ± 0.848
Integument	8.279 ± 3.005	5.709 ± 2.548	8.143 ± 4.632	5.796 ± 3.899	4.561 ± 2.709	8.593 ± 2.790
Fat	0.199 ± 0.068	0.267 ± 0.193	0.187 ± 0.123	0.247 ± 0.259	0.158 ± 0.127	0.106 ± 0.043
Muscle	6.459 ± 2.239	7.719 ± 7.781	4.857 ± 3.281	5.343 ± 3.609	3.426 ± 3.454	7.644 ± 1.683
Brain	0.151 ± 0.008	0.135 ± 0.068	0.170 ± 0.057	0.250 ± 0.125	0.240 ± 0.060	0.214 ± 0.054
Testes	0.270 ± 0.088	0.173 ± 0.088	0.203 ± 0.084	0.255 ± 0.124	0.306 ± 0.211	0.191 ± 0.080
Plasma	1.743 ± 1.006	0.805 ± 0.582	0.279 ± 0.059	0.306 ± 0.343	0.210 ± 0.142	0.263 ± 0.229
Blood-Plasma	27.409 ± 5.422	58.581 ± 26.460	65.495 ± 12.518	74.238 ± 21.427	76.763 ± 27.456	64.666 ± 7.627

The organ content at a given time point was obtained by multiplying the measured specific content (Additional file 1: Table S2, [nmoles Fe^{59} per g wet weight]) by the organ weight per body (Additional file 1: Table S4, [g per mouse]. The radioactivities are scaled to give an initial plasma value of 100. For calculation of standard deviations, see methods section "Iron content in body organs and its range of fluctuation".

The resulting dimensionless columns for each time point (measured in days) were recalculated proportionally to a tracer sum of the whole body of 100% * exp (-0.005 * t). This takes into account that the total iron content of the body is diminished at a rate of 0.5% per day [19].

The 3 slightly negative corrected measurements in spleen (see Additional file 1: Table S2), at the last three time points when the tracer content was practically zero (see Additional file 1: Table S2), were replaced by zero with common standard deviation. It was checked that this manipulation did not appreciably influence the estimated parameter values.

Table S7: Organ Fe59 Contents scaled to the whole mouse body (iron-rich diet)

Organ/Time	0.5 Day	1 Day	4 Days	7 Days	14 Days	28 Days
Duodenum	0.202 ± 0.121	0.215 ± 0.073	0.198 ± 0.163	0.050 ± 0.029	0.057 ± 0.053	0.070 ± 0.065
Liver	17.805 ± 6.146	32.613 ± 6.293	31.273 ± 16.113	17.409 ± 5.914	14.736 ± 9.756	18.488 ± 5.145
Spleen	0.699 ± 0.483	0.791 ± 0.429	0.755 ± 0.108	0.843 ± 0.430	0.597 ± 0.129	1.096 ± 0.303
Bones	36.280 ± 13.656	14.155 ± 4.663	7.174 ± 1.763	5.840 ± 2.193	7.385 ± 2.523	5.346 ± 1.280
Heart	1.780 ± 0.594	1.300 ± 0.490	1.526 ± 0.464	1.760 ± 1.063	0.567 ± 0.298	0.638 ± 0.439
Kidneys	5.409 ± 3.920	7.405 ± 2.244	6.472 ± 5.937	3.034 ± 1.739	2.103 ± 1.576	2.244 ± 1.327
Lungs	3.479 ± 3.225	1.997 ± 0.922	3.233 ± 3.436	6.736 ± 3.209	2.211 ± 2.499	1.755 ± 1.046
Stomach	0.786 ± 0.858	1.279 ± 0.619	0.674 ± 0.524	0.428 ± 0.388	0.267 ± 0.256	0.365 ± 0.262
Intestine	5.403 ± 3.506	5.408 ± 2.111	3.463 ± 2.808	1.727 ± 1.155	1.262 ± 0.602	1.304 ± 0.946
Integument	7.535 ± 4.073	9.102 ± 2.495	5.410 ± 4.243	6.744 ± 3.646	4.822 ± 2.353	6.030 ± 2.325
Fat	0.235 ± 0.132	0.320 ± 0.163	0.317 ± 0.228	0.369 ± 0.182	0.293 ± 0.079	0.105 ± 0.110
Muscle	10.188 ± 5.196	12.307 ± 4.765	9.143 ± 4.663	9.119 ± 2.432	7.245 ± 3.695	4.529 ± 2.310
Brain	0.089 ± 0.089	0.108 ± 0.006	0.080 ± 0.080	0.160 ± 0.080	0.190 ± 0.064	0.159 ± 0.009
Testes	0.455 ± 0.585	0.193 ± 0.080	0.164 ± 0.064	0.245 ± 0.083	0.227 ± 0.121	0.203 ± 0.073
Plasma	2.186 ± 0.626	0.397 ± 0.207	0.146 ± 0.056	0.083 ± 0.028	0.134 ± 0.088	0.305 ± 0.106
Blood-Plasma	7.220 ± 2.139	11.912 ± 3.895	27.992 ± 2.862	42.013 ± 6.363	51.145 ± 11.190	44.300 ± 8.961

The organ content at a given time point was obtained by multiplying the measured specific content (Additional file 1: Table S3, [nmoles Fe^{59} per g wet weight]) by the organ weight per body (Additional file 1: Table S4, [g per mouse]. The radioactivities are scaled to give an initial plasma value of 100. For calculation of standard deviations, see methods section "Iron content in body organs and its range of fluctuation".

The resulting dimensionless columns for each time point (measured in days) were recalculated proportionally to a tracer sum of the whole body of 100% * exp (-0.005 * t). This takes into account that the total iron content of the body is diminished at a rate of 0.5% per day [19].

Table S8: Absolute Flux rates along the pathways enumerated in Figure 1 (µg/body/day).

	Absolute Flux Rates (µg/body/day)		
**Parameter**	**Deficient**	**Adequate**	**Rich**
1kp_bon	15.198	19.007	13.556
2kp_kid	0.480	0.668	3.177
3kp_int	1.123	1.348	1.831
4kp_liv	2.610	3.920	10.292
5kp_sto	0.105	0.182	0.536
6kp_intg	1.190	1.716	2.602
7kp_fat	0.050	0.067	0.130
8kp_mus	1.106	2.240	4.932
9kp_lun	0.907	0.457	1.229
10kp_duo	0.021	0.064	0.075
11kp_bra	0.032	0.048	0.041
12kp_hea	0.132	0.202	0.714
13kp_tes	0.046	0.082	0.084
14kkid_p	0.480	0.668	3.177
15kliv_p	2.610	3.920	10.292
16ksto_out	0.105	0.182	0.536
17kfat_p	0.050	0.067	0.130
18kmus_p	1.106	2.240	4.932
19klun_p	0.907	0.457	1.229
20kbra_p	0.032	0.048	0.041
21khea_p	0.132	0.202	0.714
22ktes_p	0.046	0.082	0.084
23kspl_p	15.198	19.007	13.556
24kintg_out	1.190	1.716	2.602
25kint_out	1.123	1.348	1.831
26kduo_p	not calculable	not calculable	not calculable
27kbon_rbc	11.661	17.368	12.426
28kbon_spl	3.537	1.639	1.131
29krbc_spl	11.661	17.368	12.426

The values of fluxes 1 to 13 were calculated from fractional plasma clearance into the peripheral organ (table 1) times the plasma iron concentration (Additional file 1: Table S9). Parameters 14 to 22 and 24 to 25 equal the pertinent influx rates (due to steady state). Also flux 23 equals flux 1 (steady-state), so does the sum of fluxes 27 and 28. The sum of flux rates 27 into RBC and 28 directly into spleen equal flux 1 (steady-state), and the respective shares of fluxes 27 and 28 are calculated from the pertinent fractional rates (table 1). Duodenum was left out, because the tracer flux from flux in to organ does not represent total iron flux.

Table S9: Plasma iron compartment in mouse (C57B6/L) in different dietary regimes

	Iron-Deficient	Iron-Adequate	Iron-Loaded
Plasma iron concentration [µM] [1]	15.2 ± 5.5	19.8 ± 1.4	25.8 ± 1.0
scaled to [µg/dL] [2]	84.0	110.5	144.1
Plasma pool size [µg per mouse] [3]	1.15	1.50	1.96
Plasma iron turnover rate [µM/day] [4]	139	185	245
Scaled to µg per mouse[5]	11	14	19
clearance parameter [d^{-1}] [6]	17.2	18.1	18.8
Plasma half-life time of iron [min] [7]	58	55	53

Annotations:

1) Female mice, 9 weeks old, data obtained from Trinder et al. [10] Figure 2. Own measurements (Vujic-Spasic & Muckenthaler, unpublished), have somewhat higher values: 32.8 in females, 24.0 in males. Non-heme iron slowly accumulates in young mice, leading to age- and weight-related volatility of levels in different studies
2) Molecular weight of iron 55.847
3) 1.36 ml plasma per 25 mg mouse (see Additional file 1: Table S4)
4) Calculation of Turnover rate [µM per day] :
   10 [per day] * plasma concentration [µM] – 13 [µM per day].
   The slope of 10 and the intercept of -13 were estimated from Figure 5 of Trinder et al. [10].
   Rounded value of row 4 * 55.847 / 1000 *1.36 ml (body plasma volume)
5) Calculated from plasma turnover rate and plasma iron concentration, taken from Figure 5 of [10]. Turnover was measured at 2 h after administration, so assuming exponential clearance the rate constant is
   -12 * ln {1 - (turnover rate) / (plasma concentration) / 12}. This estimate is probably somewhat too low due to onset of tracer reflux. For parameter estimation we therefore rounded this preset value to 20 per day.
6) In C57Bl/6J mice: half-time = ln (2) / turnover rate constant [per day] * 24 * 60 ([min]). Brodsky et al. [53] in Swiss Webster mice report half-time of 61.7 min.

Comments:

The average plasma iron concentrations of mammals are, in spite of a considerable individual and inter-species volatility, rather similar. Typical values are [µg/dL]:
Human female: 90 ± 56 [42]
Human female: 112 ± 56 [42]
Dog 129 ± 16 [39]
Rat 123 ± 22; iron-deficient diet: 129 ± 29 [41].

Plasma iron is mainly bound to transferrin. The total pool size in man is 3.3 mg per 70 kg (median; range 1.98-5.3). This is the same range as mouse (1.5 µg/25 g would scale up to 4.2 mg per 70 kg). Rat has a plasma iron pool size (scaled to body mass) similar to mouse.

The normal flux rates out of plasma into bone marrow (assume 70 - 90% of 14 µg / d / mouse, i.e. 10-13 µg / d / mouse) reach the red blood cell pool through the intermediary process of maturation in the bone marrow. Presumably the table value is somewhat too low, again because the turnover rate of tracer does not take possible reflux into account.

Comparison with erythrocyte turnover data: This allows calculating iron flux through the erythron indirectly via the iron content of red blood cells. With 120 g/l Hb = 384 µg iron /l blood (iron content 0.032% of Hb [61]) = 800 µg/l packed erythrocytes (hematocrit, assumed 48%) one has 603.5 µg iron per 25 g mouse per day (0.71 ml red blood cell volume per mouse, see table 4; (120/0.48*1000*0.71*0.0032 = 603.5)).

The life time of mouse erythrocytes has been reported by a number of authors between 20 and 54 days [52, 60, 62-64].
If the life-time of a mouse erythrocyte is 40 – 43 days then in a across-all-ages population of cells this means a flux rate of 603.5/43 = 14 µg per mouse per day through the erythron. This is in the range of the ferrokinetic models.

Table S10: Tracer-accessible compartment sizes of the peripheral tissues (µg per mouse)

Compartment size	Fe-Deficient	Fe-Adequate	Fe-Loaded
Red blood cells	**380**	**284**	**390**
Integument	**42.2**	**39.2**	**36.4**
Liver	**10.4**	**28.4**	**99.1**
Bone marrow	**6.3**	**16.3**	**24.7**
Muscles	**35.5**	**14.5**	**35.1**
Intestinal tract	**3.7**	**3.8**	**8.4**
Kidneys	**2.4**	**3.3**	**13.6**
Spleen	**1.0**	**2.6**	**7.1**
Heart	**2.2**	**2.6**	**4.3**
Lungs	**2.2**	**2.4**	**14.3**
Brain	**1.6**	**0.8**	**1.5**
Testicles	**0.9**	**0.9**	**1.3**
Fat	**0.5**	**0.5**	**1.3**
Stomach	**0.6**	**0.5**	**1.9**

These values were calculated by dividing the influx rate into a compartment (Additional file 1: Table S8) by the pertinent clearance parameter out of the compartment (table 1).

Table S11: Residence time of molecule in compartments (days)

	Fe-Deficient	Fe-Adequate	Fe-Loaded		expressed in hours	
Plasma TF-bound*	**0.05**	**0.05**	**0.05**	**1.2**	**1.2**	**1.2**
Spleen	**0.07**	**0.14**	**0.52**	**1.6**	**3.3**	**12.6**
Bone marrow	**0.4**	**0.9**	**1.8**	**9.9**	**20.5**	**43.7**
Intestinal tract	**4.2**	**2.8**	**3.9**			
Stomach	**5.7**	**2.7**	**3.5**			
Lungs	**2.4**	**5.2**	**11.6**			
Kidneys	**5.0**	**5.0**	**4.3**			
Muscles	**32.1**	**6.5**	**7.1**			
Liver	**4.0**	**7.3**	**9.6**			
Fat	**10.3**	**7.9**	**10.1**			
Testicles	**20.5**	**10.8**	**15.0**			
Heart	**16.8**	**13.0**	**6.1**			
Red blood cells	**32.6**	**16.4**	**31.4**			
Brain	**49.7**	**16.4**	**35.7**			
Integument	**35.4**	**22.8**	**14.0**			

The expected residence time of a tracer iron molecule entering a compartment was calculated as inverse of the pertinent clearance rate constant (estimates in table 1).

Table S12: Non-heme iron content of mouse (C57B6/L) organs in different dietary regimes

Diet	Fe-Deficient	**Fe-Deficient**	Fe-Adequate	**Fe-Adequate**	Fe-Loaded	**Fe-Loaded**
	Content	**Whole organ**	Content	**Whole organ**	Content	**Whole organ**
Organ	µg/g wwt	**µg**	µg/ g wwt	**µg**	µg/g wwt	**µg**
Liver*	48.8 ± 5.1	**59.5 ± 7.9**	86.7 ± 14.9	**106 ± 20**	1310 ± 90	**1600 ± 170**
Kidneys	36.9 ± 2.3	**14.0 ± 2.0**	60.8 ± 2.4	**23.1 ± 3.2**	88.1 ± 7.1	**33.5 ± 5.2**
Spleen	141 ± 46	**9.9 ± 3.5**	254 ± 99	**17.8 ± 7.4**	1760 ± 240	**123 ± 24**
Heart	71.2 ± 2.4	**10.0 ± 1.5**	68.2 ± 7.9	**9.5 ± 1.8**	81.8 ± 10.5	**11.5 ± 2.2**
Duodenum				**2.9 ± 0.3**		
Lung				**9.5 ± 0.8**		
Brain				**13.4 ± 1.7**		
Muscle				**142 ± 29**		

* Frazer et al. [65] give 56 µg/g wwt. Vácha et al. [66] report 257 µg/g wwt.

The concentration (µg non-heme iron per g of organ) data are unpublished own data (K.Schümann for liver, kidneys, spleen and heart) and Vujic-Spasic & Muckenthaler for duodenum, lung, brain and muscle. The whole organ data were calculated as concentration * organ mass. The standard deviation was estimated from that of both sets of measurements (see methods). Note that liver, kidney and spleen store iron (ferritin) with increasing supply, whereas heart does not.

The non-heme iron content of organs (normal guinea pigs, Rebouche et al. [67]) is about 80% in liver, 62% in kidney and 34% in heart (related to total iron content, but apparently not corrected for blood content). Vácha et al. [66] give 12.2 µg/g spleen (64% of its total iron).

According to Lee et al. [54] the spleen contains 5.25% of all macrophages (measured by specific monoclonal antibody), whereas 16% of this total macrophage pool is in the liver as Kupffer cells. If non-heme iron concentration of spleen and of Kupffer cells would be the same, then about 50 % of the liver non-heme iron would be in Kupffer cells (calculated example in adequate case: 17.8*16/5.25/106) in the adequate and deficient and about 25% in the loaded regime. However, van Wyk et al. ([48], their table 2) found nearly all non-heme iron in hepatocytes (in control rats) and only in rats after dextran-iron loading about 28% of the accumulated iron in Kupffer cells.

Table S13: Tracer-accessible Iron Pools compared with non-heme Iron in different dietary Regimes

Diet	Fe-Deficient	Fe-Deficient	Fe-Adequate	Fe-Adequate	Fe-Loaded	Fe-Loaded
	Content	% of non-heme iron	Content	% of non-heme iron	Content	% of non-heme iron
Organ	µg per mouse		µg per mouse		µg per mouse	
Liver	10.4	17	28.4	27	99.1	6
Kidneys	2.4	17	3.3	14	13.6	41
Spleen	1.0	10	2.6	15	7.1	6
Heart	2.2	22	2.6	27	4.3	37

The tracer-accessible pool sizes were calculated as described in Additional file 1: Table S10 (see visualization in Additional file 2: Figure S4). For the "cold" non-heme iron content refer to Additional file 1: Table12.

It may be noted that upon iron loading non-heme iron in liver and spleen is less accessible to tracer uptake, whereas in kidneys and heart the tracer-reachable fraction increases.
