# Supplementary material for: Systems analysis of iron metabolism: the network of iron pools and fluxes
Source: BMC Syst Biol. 2010 Aug 13;4:112. doi: 10.1186/1752-0509-4-112 (PMC2942822; doi:10.1186/1752-0509-4-112)
Supplement: Additional file 2 — This file contains 4 supplementary figures describing graphically our model layout and some information mentioned in the manuscript text. [file 1752-0509-4-112-S2.PDF]

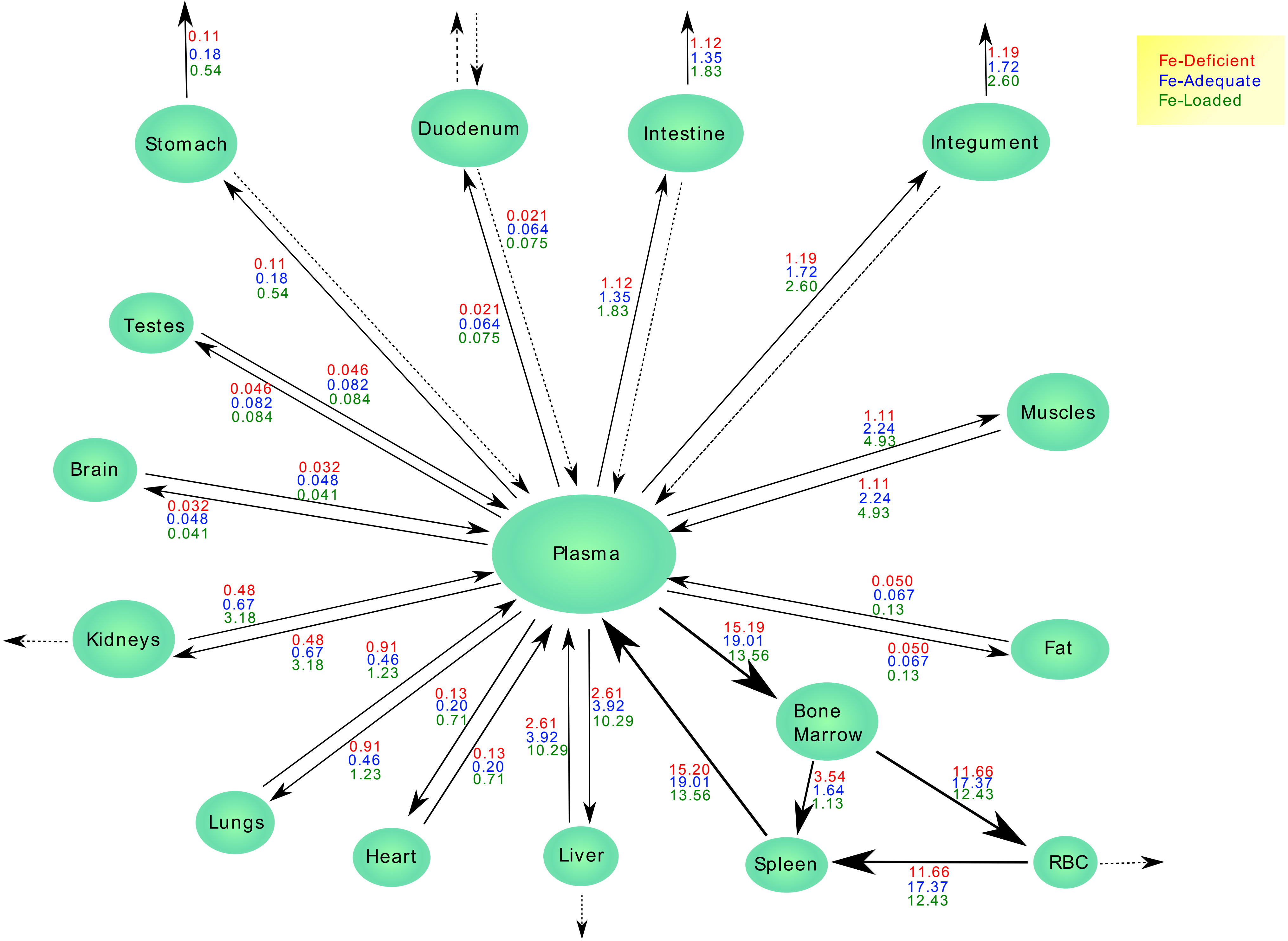

Figure S1: Turnover rates of iron flux from plasma into periphery and of return fluxes and/or loss of iron from the periphery
This figure summarizes the main flux estimates from the ferrokinetic data (supplementary table 8). They apply to the three different dietary conditions (iron-deficient – red; iron-adequate – blue; iron-loaded – green), expressed as μg per mouse per day.

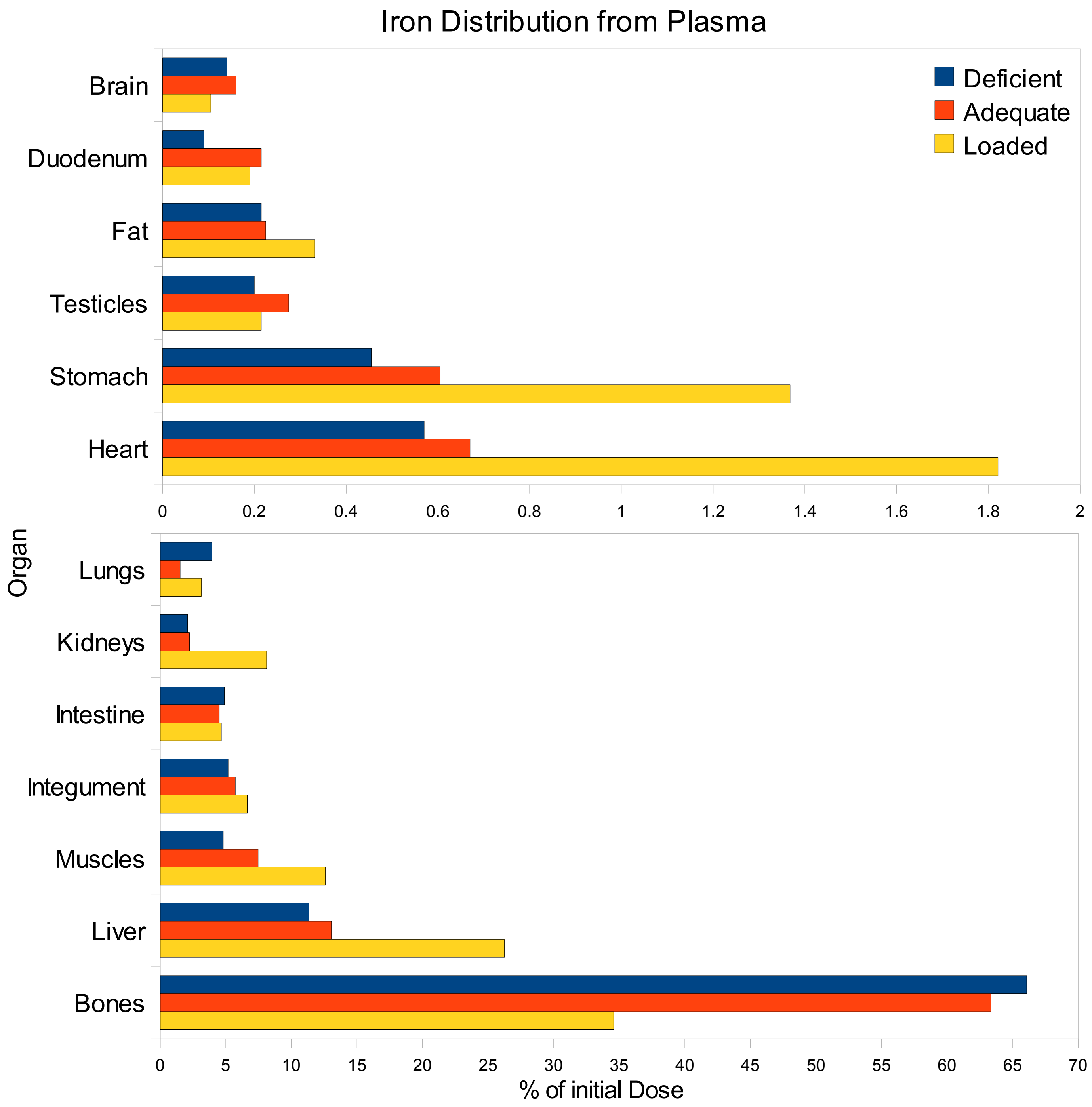

Figure S2: Iron Distribution into the Peripheral Compartments

This column diagram shows the relative share of peripheral compartments of the transferrin-bound iron leaving the central plasma / Extravascular Fluid (EVF) compartment. It is expressed as % of the initial tracer dose.

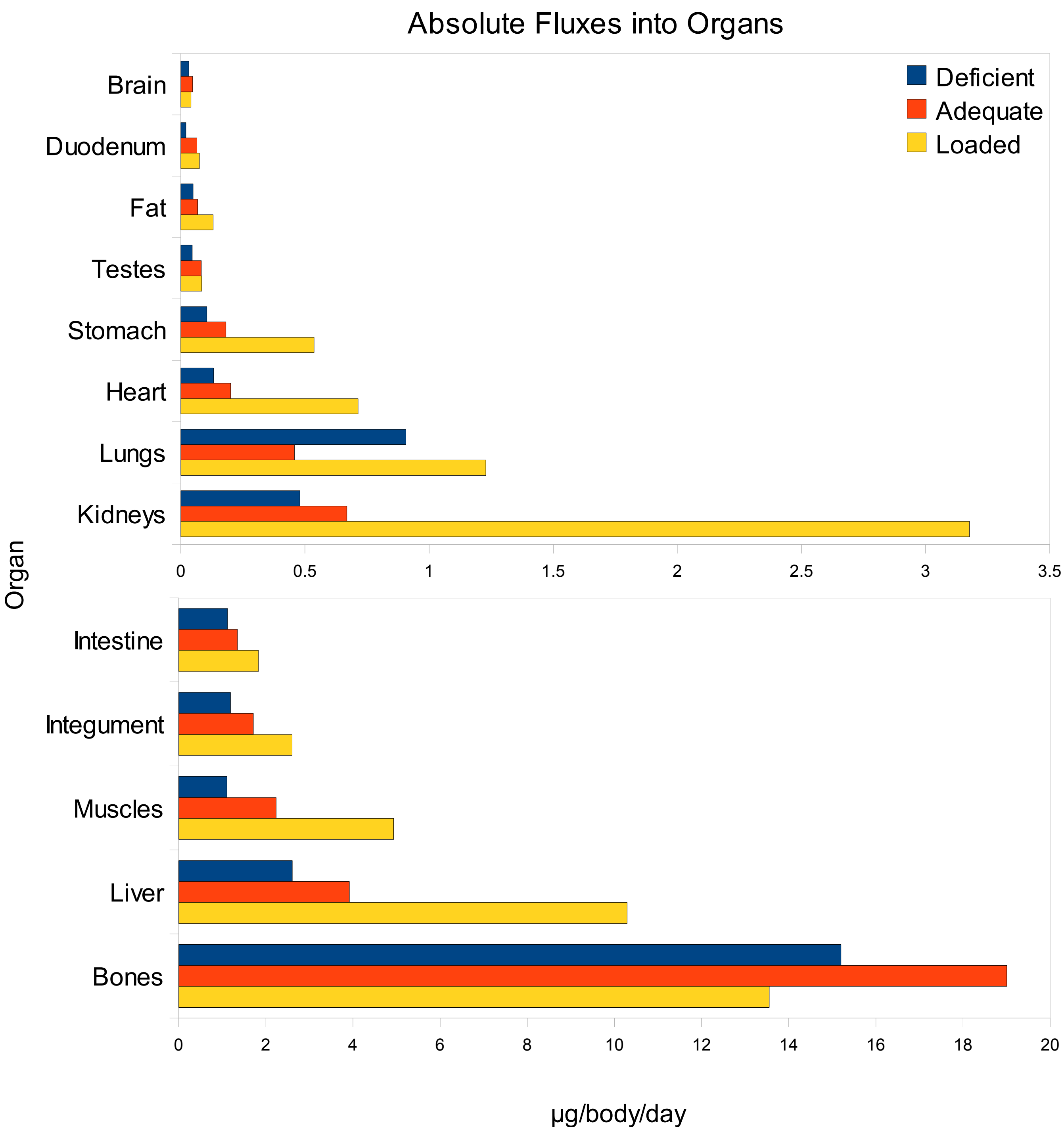

Figure S3: Iron Turnover Rate in the Peripheral Compartments

This flux rate (µg per day per 25 g mouse) was calculated from plasma iron content (supplementary table 9) and the fractional clearance rate into the respective compartment (table 1). Note the two scales.

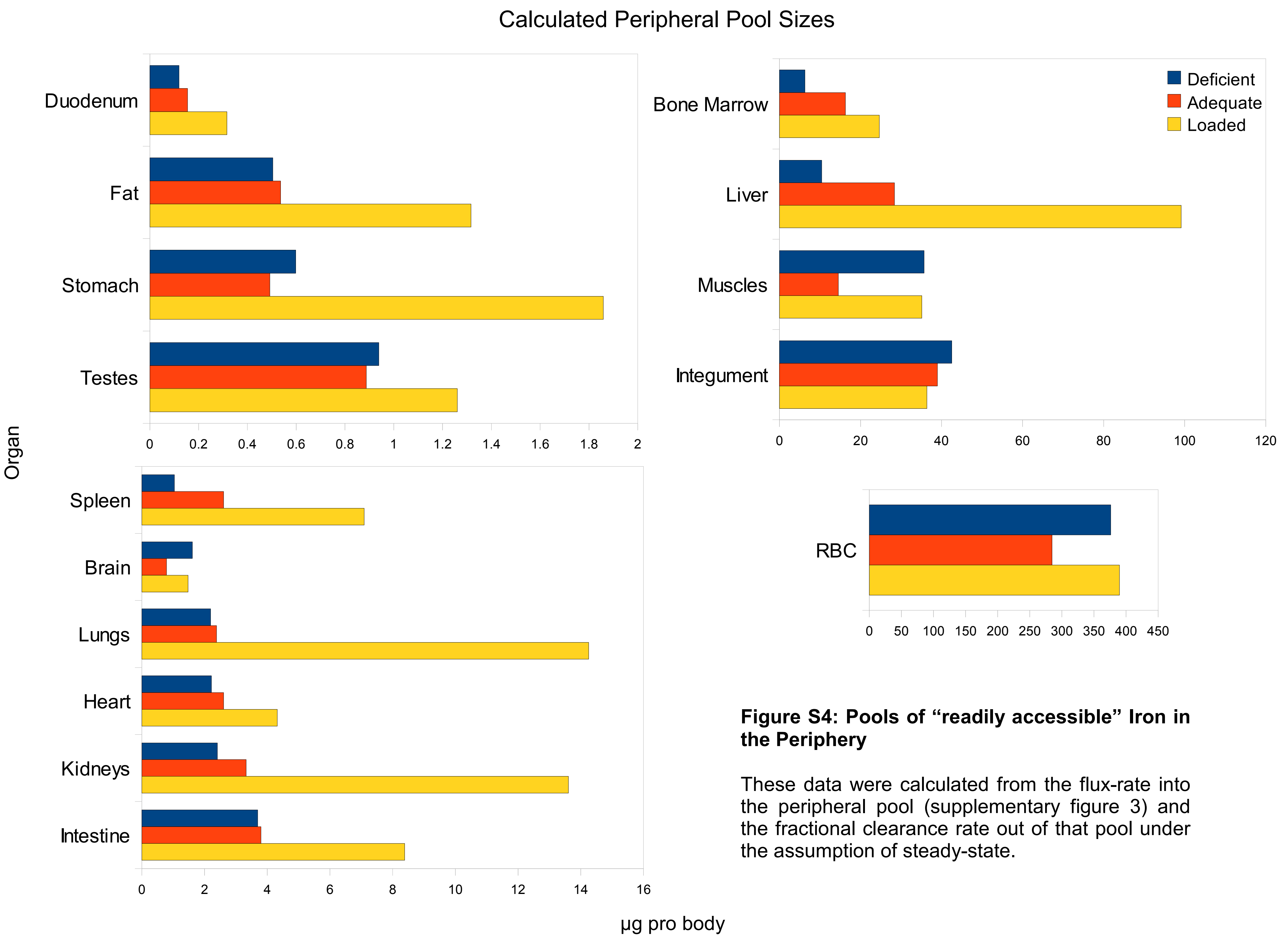

Figure S4: Pools of "readily accessible" Iron in the Periphery

These data were calculated from the flux-rate into the peripheral pool (supplementary figure 3) and the fractional clearance rate out of that pool under the assumption of steady-state.
