# Supplementary material for: Systems analysis of iron metabolism: the network of iron pools and fluxes
Source: BMC Syst Biol. 2010 Aug 13;4:112. doi: 10.1186/1752-0509-4-112 (PMC2942822; doi:10.1186/1752-0509-4-112)
Supplement: Additional file 3 — This has file has the differential equations that compose our mathematical model. [file 1752-0509-4-112-S3.DOC]

d(Plasma) / dt = Plasma(t) * (-1kp_bon - 2kp_kid - 3kp_int - 4kp_liv - 5kp_sto -6kp_intg

– 7kp_fat - 8kp_mus - 9kp_lun -10kp_duo - 11kp_brain

- 12kp_hea - 13kp_tes)

+14kkid_p * Kidneys(t) + 15kliv_p * Liver(t) + 16ksto_out * Stomach(t)

+ 17kfat_p * Fat(t) + 18kmus_p * Muscle(t) + 19klun_p * Lungs(t)

+ 20kbra_p * Brain(t) + 21khea_p * Heart(t) + 22ktes_p * Testes(t)

+ 23kspl_p * Spleen(t)

d(Bone Marrow) / dt = 1kp_bon * Plasma(t) -Bone Marrow(t) * ( 27kbon_rbc + 28kbon_spl)

d(Liver) / dt = 4kp_liv * Plasma(t) - 15kliv_p * Liver(t)

d(Spleen) / dt = - 23kspl_p * Spleen(t) + 29krbc_spl * RBC(t) + 28kbon_spl * Bone Marrow(t)

d(Heart) / dt = 12kp_hea * Plasma(t) - 21khea_p * Heart(t)

d(Testes) / dt = 13kp_tes * Plasma(t) - 22ktes_p * Testes(t)

d(Lungs) / dt = 9kp_lun * Plasma(t) - 19klun_p * Lungs(t)

d(Kidneys) / dt = 2kp_kid * Plasma(t) - 14kkid_p * Kidneys(t)

d(Muscle) / dt = 8kp_mus * Plasma(t) - 18kmus_p * Muscle(t)

d(Fat) / dt = 7kp_fat * Plasma(t) - 17kfat_p * Fat(t)

d(Integument) / dt = 5kp_intg * Plasma(t) - 24intg_out * Integument(t)

d(Duodenum) / dt = 10kp_duo * Plasma(t) - 26kduo_p * Duodenum(t)

d(Stomach) / dt = 5kp_sto * Plasma(t) - 16ksto_out * Stomach(t)

d(RBC) / dt = 27kbon_rbc * Bones(t) -29krbc_spl * RBC(t)

d(Intestine) / dt = 3kp_int * Plasma(t) - 25int_out * Intestine(t)

d(Brain) / dt = 11kp_brain * Plasma(t) - 20kbra_p * Brain(t)

d(Outside) / dt = 25int_out * Intestine(t) + 16ksto_out * Stomach(t)
 + 24intg_out * Integument(t)
